# Profound cell wall remodeling in *Candida parapsilosis* during systemic infection confers simultaneous tolerance to echinocandins and host immunity

**DOI:** 10.1128/spectrum.03043-25

**Published:** 2026-02-13

**Authors:** Farnaz Daneshnia, Liuyang Cai, Deepika Gunasekaran, Isha Gautam, Austin M. Perry, Pegah Mosharaf Ghahfarokhy, Arefeh Ebadati, Julieta Munoz, Dorian Padilla, Süleyha Hilmioglu-Polat, Shenglin Mei, Daniel J. Floyd, Miquel Àngel Schikora-Tamarit, Diego Fuentes, Maria Artigues-Lleixà, Louise A. Walker, Samuel M. Gonçalves, Mostafa Salehi, Agostinho Carvalho, Jigar V. Desai, David S. Perlin, Carol A. Munro, Alex Hopke, Tuo Wang, Toni Gabaldón, Wenjie Fang, Clarissa J. Nobile, Michael K. Mansour, Amir Arastehfar

**Affiliations:** 1 Institute of Biodiversity and Ecosystem Dynamics (IBED), University of Amsterdam 1234, Amsterdam, Netherlands; 2 Division of Infectious Diseases, Massachusetts General Hospital 2348, Boston, Massachusetts, USA; 3 Department of Dermatology, Shanghai Key Laboratory of Molecular Medical Mycology, Shanghai Changzheng Hospital, Naval Medical University 12521, Shanghai, China; 4 The Center for Fungal Infectious Diseases Basic Research and Innovation of Medicine and Pharmacy, Ministry of Education, Shanghai, China; 5 Department of Molecular and Cell Biology, School of Natural Sciences, University of California Merced 33244, Merced, California, USA; 6 Department of Chemistry, Michigan State University 242469, East Lansing, Michigan, USA; 7 Quantitative and Systems Biology Graduate Program, University of California 33244, Merced, California, USA; 8 Health Sciences Research Institute, University of California 33244, Merced, California, USA; 9 Department of Medical Microbiology, Ege University Faculty of Medicine 60521, Izmir, Turkey; 10 Fralin Biomedical Research Institute, Virginia Tech FBRI21 Cancer Research Center 145763 , Washington, DC, USA; 11 Department of Biomedical Sciences and Pathobiology, College of Veterinary Medicine, Virginia Tech 1757, Blacksburg, Virginia, USA; 12 Life Sciences Programme, Supercomputing Center (BSC-CNS) 132144 , Barcelona, Spain; 13 Institute for Research in Biomedicine (IRB Barcelona), The Barcelona Institute of Science and Technology 518635, Barcelona, Spain; 14 School of Medicine, Medical Sciences and Nutrition, Institute of Medical Sciences, University of Aberdeen 150676, Aberdeen, United Kingdom; 15 School of Medicine, Life and Health Sciences Research Institute (ICVS), University of Minho 224744, Braga, Portugal; 16 ICVS/3B’s-PT Government Associate Laboratory, Guimarães, Braga, Portugal; 17 Department Industrial Engineering Faculty of K.N, KN Toosi University of Technology 108871, Tehran, Iran; 18 Center for Discovery and Innovation, Hackensack Meridian Health, Nutley, New Jersey, USA; 19 Georgetown University Lombardi Comprehensive Cancer Center 66634, Washington, DC, USA; 20 Department of Medical Sciences, Hackensack Meridian School of Medicine 576909, Nutley, New Jersey, USA; 21 Department of Biomedical Sciences, East Tennessee State University Quillen College of Medicine, Center of Excellence in Inflammation, Infectious Disease and Immunity, East Tennessee State University 248026, Johnson City, Tennessee, USA; 22 Catalan Institution for Research and Advanced Studies 117370, Barcelona, Spain; 23 Centro de Investigación Biomédica en Red de Enfermedades Infecciosas (CIBERINFEC), Barcelona, Spain; 24 Department of Medicine, Harvard Medical School 205260 , Boston, Massachusetts, USA; 25 University Hospital Wuerzburg, Medical Hospital II, Würzburg, Germany; Universita degli Studi di Modena e Reggio Emilia, Modena, Italy

**Keywords:** echinocandins, polyenes, macrophage polarization, biofilms, cell wall remodeling, resistance, tolerance, immune evasion

## Abstract

Antifungal tolerance can promote the emergence of resistance yet often incurs fitness costs for fungal pathogens. How tolerant populations compensate for these deficits and how they may be therapeutically targeted remain poorly understood. Here, we investigate four sequential *Candida parapsilosis* isolates recovered from a patient with persistent candidemia and failure of micafungin therapy. The infection was ultimately cleared with liposomal amphotericin B (LAMB). Whole-genome sequencing (WGS) confirmed clonal relatedness and the absence of known resistance mutations. Later isolates displayed marked cell wall remodeling (CWR), characterized by increased mannan and reduced β-glucan content, as revealed by microscopy and solid-state nuclear magnetic resonance. These isolates formed thicker biofilms and displayed enhanced echinocandin tolerance but paradoxically showed increased susceptibility to amphotericin B (AMB) *in vitro* and during systemic infection in mice. Despite a complex mutational landscape, transcriptomic profiling across planktonic and biofilm growth showed minimal divergence from the earliest isolate. Functionally, evolved isolates suppressed M1 macrophage polarization, dampened proinflammatory cytokine production, survived better during neutrophil interactions, and transiently increased fungal burden *in vivo*. These findings show that host-driven CWR could promote echinocandin tolerance while simultaneously sensitizing *C. parapsilosis* to AMB. Our results suggest that alternating echinocandin and LAMB therapy may effectively eliminate echinocandin-tolerant fungal populations.

**IMPORTANCE:**

Antifungal tolerance is increasingly recognized as a precursor to resistance, yet its clinical and biological consequences remain poorly defined. By analyzing sequential *Candida parapsilosis* isolates from a case of persistent candidemia, we show that cell wall remodeling is associated with echinocandin tolerance, alters host immune interactions, and increases susceptibility to amphotericin B (AMB). These findings reveal how tolerance-associated adaptations shape pathogen fitness during infection and highlight the therapeutic potential of alternating echinocandin and AMB therapy. This work advances our understanding of antifungal tolerance and suggests that exploiting opposing drug susceptibilities may improve treatment outcomes for challenging-to-treat *Candida* infections.

## INTRODUCTION

*Candida parapsilosis* is a leading cause of candidemia worldwide, particularly among neonates, immunocompromised individuals, and patients with intravascular devices (1). Its capacity to form robust biofilms enables persistence on medical devices and hospital surfaces, promoting nosocomial transmission and reduced susceptibility to antifungal drugs (1–3). Historically, *C. parapsilosis* outbreaks were sporadic and involved drug-susceptible isolates. However, since ~2015, fluconazole-resistant (FLCR) isolates have driven persistent outbreaks across numerous regions, particularly in low- and middle-income countries where fluconazole remains a first-line agent (1–3). More recent reports from Europe (4–6), the United States (7), and Canada (8) indicate increasing detection of FLCR isolates, often associated with prolonged healthcare transmission and elevated patient mortality (9–11). In several cases, environmental strains were genetically identical to patient isolates, underscoring efficient environmental persistence and patient-to-surface-to-patient spread (9). Of growing concern, multidrug-resistant (MDR) and sporadic echinocandin-resistant *C. parapsilosis* isolates have now been documented (12–15). Unlike the historical paradigm that multidrug resistance attenuates virulence (16), MDR *C. parapsilosis* strains retain competitive fitness and virulence *in vitro* and *in vivo* (13). This raises concern that MDR *C. parapsilosis* could pose a global threat similar to the widespread emergence of FLCR isolates. Accordingly, the World Health Organization has designated *C. parapsilosis* a high-priority fungal pathogen (17).

These findings highlight the need to define mechanisms that enable *C. parapsilosis* to survive antifungal exposure and host immunity. Sequential clinical isolates and experimental evolution studies have shown that fungi readily evolve phenotypic plasticity and genetic adaptations during infection. For example, *Candida glabrata* and *Candida albicans* populations evolved within macrophages acquire filamentation ability and enhanced fitness (18, 19), while gastrointestinal passaging of *C. albicans* selects yeast-locked strains with improved colonization capacity (20). *Candida auris* isolates recovered from patients and mice frequently exhibit aggregative phenotypes driven by adhesin gene amplification or cell division defects, promoting persistence in skin and invasive niches (21, 22).

Adaptation to antifungal therapy can occur through resistance mutations or tolerance mechanisms. For example, *C. glabrata FEN1* mutations confer echinocandin tolerance (23), mitochondrial loss enhances tolerance and fluconazole resistance (24), and chromosomal aneuploidy contributes to azole tolerance in *C. albicans*, *C. parapsilosis*, and *Cryptococcus* species (13, 25–28). Although antifungal-tolerant strains can drive breakthrough infections (1), tolerance is often associated with fitness defects in drug-free conditions (23–25). Consistent with this, mixed-genotype infections in *C. albicans* demonstrate dynamic selection: virulent genotypes dominate without drug pressure, whereas tolerant genotypes expand under antifungal therapy (29). This model suggests that tolerance trades off with virulence, enabling immune clearance in the absence of drug pressure. However, it remains unclear how tolerant isolates can simultaneously withstand host defenses and antifungal drugs during systemic infection. Moreover, most insights originate from murine models; studies of serial isolates from human infection may reveal clinically relevant adaptations not captured experimentally.

To address this gap, we examined four sequential *C. parapsilosis* bloodstream isolates collected from a patient with persistent candidemia during echinocandin treatment (10–13, 30, 31). Whole-genome sequencing (WGS) confirmed clonal relatedness. Later isolates exhibited marked cell wall remodeling (CWR) and robust biofilm formation, accompanied by enhanced echinocandin tolerance *in vitro* and *in vivo*. Transcriptomic analysis of planktonic and biofilm conditions did not reveal divergent transcriptional programs. Notably, these evolved isolates did not display fitness defects; instead, they suppressed proinflammatory cytokine production, impaired M1 macrophage polarization, survived interactions with innate immune cells, and generated transiently higher fungal burdens in mice. Intriguingly, this adaptation was associated with increased susceptibility to amphotericin B (AMB).

Collectively, our findings reveal that *C. parapsilosis* can undergo profound CWR during infection that is associated with increased echinocandin tolerance while maintaining or even enhancing fitness against host immunity. These results provide mechanistic insights into fungal adaptation during persistent infection and suggest therapeutic opportunities to exploit trade-offs associated with antifungal tolerance.

## MATERIALS AND METHODS

### Kinetic growth analysis

Overnight cultures of *C. parapsilosis* isolates grown in yeast peptone dextrose (YPD) broth were washed with phosphate-buffered saline (PBS), adjusted to an OD_600_ of 0.2, and inoculated into the desired medium. A volume of 200 µL of the inoculated medium was dispensed into each well of a 96-well plate. Plates were sealed with Breathe-Easy membrane covers (Millipore Sigma) and incubated at 37 °C in a SpectraMax i3x multimode plate reader (VWR) without shaking. OD measurements were recorded hourly throughout the experiment.

### Cell wall staining of mannan, chitin, and glucan

*C. parapsilosis* isolates were cultured in YPD at 30°C for 6 h. Cells were washed three times with PBS and blocked for 30 min with fluorescence-activated cell sorting (FACS) block solution (0.5% bovine serum albumin [BSA], 5% heat-inactivated rabbit serum, 5 mM EDTA, and 2 mM NaN₃ in PBS). Following blocking, pellets were collected and washed three times with FACS buffer (0.5% BSA, 5 mM EDTA, and 2 mM NaN₃ in PBS) at 4°C. The cell suspension was then incubated with 5 µg/mL Fc:Dectin-1 protein for 1 h at 4 °C, washed three times with FACS buffer, and stained with anti-human Fc Alexa Fluor 488 (1:200) for 45 min at 4°C. After three PBS washes, cells were stained with 50 µg/mL Wheat Germ Agglutinin–Alexa Fluor 680 (to detect exposed chitin) for 30 min at 4°C, washed, and subsequently stained with 50 µg/mL Concanavalin A–Texas Red (to detect mannan) for 30 min at 4°C. All samples were imaged using differential interference contrast and fluorescence microscopy on an UltraVIEWVoX spinning disk confocal microscope (Nikon, Surrey, UK). Mannan, chitin, and β1,3-glucan exposure were quantified by measuring fluorescence intensities for individual yeast cells, with mean values calculated from 30 cells per isolate.

### High-pressure freezing transmission electron microscopy (HPF-TEM)

*C. parapsilosis* isolates were cultured in YPD at 30°C for 6 h, washed three times with PBS, and concentrated into a paste by resuspending in residual liquid. Samples were high-pressure frozen using an EMPACT2 high-pressure freezer with a rapid transfer system (Leica Microsystems, Milton Keynes, UK), followed by freeze substitution in 1% (wt/vol) OsO₄ in acetone with a Leica EMAFS2. After substitution, samples were embedded in epoxy resin with additional vacuum-assisted infiltration at 60°C and then polymerized at 60°C for 48 h. Ultrathin sections (60 nm) were prepared using a Diatome diamond knife on a Leica UC6 ultramicrotome, stained with uranyl acetate and lead citrate, and imaged with a Philips CM10 transmission electron microscope (FEI, Cambridge, UK) equipped with a Gatan Bioscan 792 (Gatan, Abingdon, UK). ImageJ was used to measure the thickness of the inner (chitin and glucan) and outer cell wall by averaging measurements for 30 cells for each *C. parapsilosis* isolate.

### Solid-state nuclear magnetic resonance (NMR)

The four *C. parapsilosis* isolates (Cp9, Cp10, Cp11, and Cp12) were cultured in Yeast Nitrogen Base medium without amino acids and ammonium sulfate but supplemented with 6.0 g/L of ^13^C glucose and 10.0 g/L of ^15^N-ammonium sulfate (Cambridge Isotope Laboratories) for uniform isotope labeling. Cultures were incubated for 3 days at 30°C. After growth, the fungal materials were harvested, washed four times, and 35 mg of the whole cell materials was packed into 3.2-mm magic angle spinning (MAS) rotors for analysis. Solid-state NMR experiments were performed on an 800 MHz (18.8 Tesla) Bruker Avance Neo spectrometer housed at the Michigan State University Max T. Roger NMR facility, with a 3.2 mm HCN MAS probe at 15 kHz MAS and 290 K. ^13^C chemical shifts were referenced to adamantane CH_2_ peak at 38.48 ppm (tetramethysilane scale). The composition of rigid and mobile components was determined by the analysis of peak volumes in 2D ^13^C-^13^C spectra using 53 ms CORD (32, 33) and DP refocused *J*-INADEQUATE schemes (34, 35). Peak volumes were extracted using Bruker Topspin software, considering only well-resolved signals to minimize spectral crowding, and the estimations of the rigid and mobile molecules were calculated as described in recent studies (36, 37). The complete assignments of the glucan peaks for the rigid polysaccharides and the mobile polysaccharides are listed in Fig. 4B and C. All spectral processing and peak integrations were performed using TopSpin 4.1.4, calculations for relative abundance were conducted in Excel, and graphical representations were generated using Origin 2021.

### Macrophage isolation, infection, and functional analysis

Leukopaks of healthy donors attending Massachusetts General Hospital (IRB# 2014P002377) were used for monocyte isolation. Leukocytes were isolated by overlaying the leukopak on Ficoll (ThermoFisher), followed by isolation and treating monolayers with red blood cell lysis buffer (STEMCELL Technologies). Monocytes were isolated using the EasySep Direct Human Monocyte Isolation Kit (STEMCELL Technologies). Viability (staining with 7-AAD) and purity (CD45, CD16, and CD14) of monocytes were analyzed using flow cytometry, as described previously (38). The viability and purity (CD14^+^-CD16^—^CD45^+^) of monocytes were ≥99% and ≥93%, respectively. To differentiate monocytes to fully mature macrophages, monocytes (3 × 10^5^/well) were seeded in 24-well plates in fresh complete RPMI=cRPMI (10% heat-inactivated FBS, 1% pen-strep, and 1% L-glutamine) supplemented with 50 ng/mL of M-CSF. Macrophages were washed with PBS on day 5 and treated with cRPMI containing 50 ng/mL of M-CSF. Mature macrophages were infected with *C. parapsilosis* isolates on day 8. The overnight-grown *C. parapsilosis* isolates in fresh YPD broth were washed with PBS, and the colony-forming units (CFUs) were adjusted in accordance with the experiment.

To determine *C. parapsilosis* survival, macrophages were infected with a multiplicity of infection (MOI) of 3 yeasts/1 macrophage (3/1), followed by incubation at a CO_2_ incubator, washing at 1 h post-infection, and macrophage lysis at designated time points using ice-cold water, as described previously (38). Survival was determined by normalizing CFUs at designated time points against the 1-h time point. Phagocytosis was determined using flow cytometry. Briefly, macrophages stained with CellMask-Deep Red (ThermoFisher) were infected with an MOI of 5/1 FITC-stained *C. parapsilosis* isolates. Infected macrophages were washed with PBS at 1 h post-infection, detached from wells by treating with ACCUTASE (STEMCELL Technologies), and subjected to flow cytometry and determination of the mean fluorescence intensity (MFI) of the double-positive events. Macrophage reactive oxygen species (ROS) was determined by infecting macrophages (stained with CellMask-Deep Red [ThermoFisher]) with *C. parapsilosis* (MOI = 5). Macrophages were washed with PBS at 1 h post-infection, followed by treating with fresh cRPMI and further incubation in a CO_2_ incubator. Macrophages were treated with dihydroxyrhodamine 1,2,3 (ThermoFisher) 135 min post-infection, and macrophages were washed and detached using ACCUTASE at 3 h post-infection. Next, macrophages were subjected to flow cytometry, and the ROS levels (MFIs) of stained macrophages (APC700) were determined. Uninfected macrophages were used for gating.

### Cytokine measurement

Primary human macrophages were infected with *C. parapsilosis* isolates at an MOI = 10. Supernatants were collected 24 h post-infection, subjected to cytokines, and were quantified using ELISA MAX Deluxe set Kits (BioLegend) in accordance with the manufacturer’s instructions (39).

### Neutrophil isolation and infection

Fresh neutrophils were isolated from the blood of healthy donors (IRB #2014P002377), and primary human neutrophils were isolated using the EasySep Human Neutrophil Isolation Kit (STEMCELL Technologies). The viability and purity of neutrophils were assessed as discussed previously and were always ≥99%. Freshly isolated neutrophils were co-incubated with *C. parapsilosis,* and the MOI varied depending on the experiment. ROS production, phagocytosis, and killing were determined as outlined previously (13).

Neutrophil swarming and analysis were carried out as previously described (40).

### Biofilm crystal violet and confocal scanning laser microscopy (CSLM) assays

All biofilm analyses were performed as described previously (13).

### Antifungal tolerance of biofilms

*C. parapsilosis* isolates were grown overnight in YPD broth, centrifuged, and washed with PBS. Then, 1 mL of the adjusted cells (OD_600_ = 1 in 1 mL YPD broth) was added into 24-well plates (1 mL/well), followed by incubation at 37°C for 24 h. After incubation, the wells were extensively washed with PBS to remove non-adherent cells, followed by adding 1 mL of RPMI containing desired concentrations of antifungal drugs. Untreated control samples were used for normalization purposes. *C. parapsilosis* cells were washed with PBS at designated time points, treated with PBS containing sublethal proteinase K concentrations (20 µg/mL), and incubated at 37°C for 30 min, followed by detaching cells from the wells using a sterile scraper. The collected cells were plated on YPD for CFU determination, and viability was determined by normalization against pertinent untreated controls.

### Systemic infection mouse models

Systemic infection mouse models were carried out according to the previously established protocol (13). Briefly, mice were infected with 2 × 10^7^ CFU of each isolate using tail vein injection and sacrificed on designated time points, followed by harvesting and homogenizing organs and plating on YPD agar plates for CFU counting. The CFUs obtained from each organ were normalized against the CFUs of the pertinent initial inoculum used to infect mice. For experiments involving antifungal treatment, mice received desired antifungal drugs 24 h post-infection and were treated every other day (5 mg/kg for both caspofungin and AMB). Five mice were included for each isolate at each time point. CFUs of organs at the desired time point were normalized against the pertinent untreated control mice, and results were presented as percentages.

### RNA extraction, library preparation, and sequencing

Growth of *C. parapsilosis* biofilms was performed as described previously (41). Biofilms were harvested by gently pipetting up and down along the bottoms of the 12-well plates and combining the biofilm slurry of the same strain from each well of one 12-well plate into a 50 mL conical tube. Planktonic cultures of a given biological replicate were combined in a 50 mL conical tube. Three biological replicates were completed for each sample and each time point. The conical tubes were centrifuged at 4,000 × *g* for 5 min, and the supernatant was aspirated. Pellets were snap-frozen and stored at −80°C until RNA extraction. Total RNA was extracted using the Ribopure RNA Purification Kit for Yeast (AM1926). mRNA was separated from total RNA using the NEBNext Poly(A) mRNA Isolation Module (E7490L). Then, 500 ng of purified mRNA was made into cDNA libraries using the NEBNext Ultra II RNA Library Prep Kit for Illumina (E7775L). Libraries were sequenced on the AVITI 150 PE75 at the UC Davis Sequencing Core.

### RNA-seq data processing

Paired-end reads were assessed for quality using FastQC (v0.11.9) (42). The reference *C. parapsilosis* genome and its annotation were retrieved from the *Candida* Genome Database (CGD) (43) on 16 January 2024. The reference genome was indexed using the subread-buildindex function in subread (v2.0.6) (44). We then mapped the paired-end reads to the reference genome using the subread-align function in subread (v2.0.6) (44), including multi-mapped reads, mapping to the best 10 genomic loci (parameters: –multiMapping, -B 10). Transcript abundance for each gene was computed using the featureCounts function in subread (v2.0.6) (44), counting only read-pairs where both ends map to the same chromosome (parameters: -B, -C, –countReadPairs). The transcript abundance table was generated by consolidating all serial isolates, biological replicates, time points (90 min, 8 h, and 24 h), and growth conditions (biofilm and planktonic) using a custom Python script (v3.8.18) and was used for subsequent differential expression analyses.

### Differential expression and functional enrichment analyses of the *C. parapsilosis* transcriptome

Transcript abundances were used to identify differentially expressed genes in the four serial isolates (Cp9, Cp10, Cp11, and Cp12) across three time points (90 min, 8 h, and 24 h) and two growth conditions (biofilm and planktonic). Differential expression analysis was performed using DESeq2 (v1.44.0) (45). We subset samples to each time point and estimated size factors independently for each time point. The design matrix was constructed to get differential expression between conditions, strains, and strain-specific effect on growth condition using an interaction term. *P*-value estimates were adjusted for multiple hypothesis testing using independent hypothesis weighting (v1.32.0) (46), and log2 fold change shrinkage was performed using apeglm (v1.26.1) (47). For the heatmap comparing expression of cell wall synthesis genes, we obtained genes in *S. cerevisiae* involved in this process and identified corresponding orthologs in *C. parapsilosis* as annotated in CGD (43). For the heatmap related to biofilm genes and transporters, *C. albicans* genes with Gene Ontology (GO) functions “single-species biofilm formation on inanimate substrate” and “xenobiotic transmembrane transporter activity” were obtained from CGD along with their corresponding *C. parapsilosis* orthologs (43). The heatmaps were generated using the pheatmap package (v1.0.12) in R (v4.4.3). Functional enrichment of gene categories across isolates was performed using Gene Set Enrichment Analysis (GSEA) (48) by sorting genes in their descending order of expression. GSEA was performed using the clusterProfiler package (v4.12.6) in R (v4.4.3).

### Bulk RNA-Seq data processing and enrichment analysis of the macrophage transcriptome

Raw gene-level count data were processed in R. Expression counts were normalized, and differential expression analysis was conducted using DESeq2 (45). For each time point (3 h and 24 h), pairwise comparisons were performed across isolates (e.g., Cp10 vs Cp9, Cp11 vs Cp9, and Cp12 vs Cp10). Moderated log2 fold-change estimates were obtained using DESeq2’s lfcShrink() function with the “ashr” method (49). Genes were ranked by fold change and used as input for GSEA (48). GSEA was performed using the fgseaMultilevel() function from the fgsea R package (48); https://www.biorxiv.org/content/10.1101/060012v3), with gseaParam = 2. Enrichment was tested against MSigDB’s Hallmark, GO:BP, and Canonical pathway collections, as well as a custom macrophage polarization gene set derived from Xue et al. (50). Enrichment curves were generated using the plotEnrichment() function, and ranked normalized enrichment score (NES) scatter plots were used to highlight the most significantly enriched pathways in each comparison.

### Variant calling

We used perSVade (v0.8) (51) to call and filter variants from raw sequencing data for each strain (see the supplemental material for details). In brief, the pipeline was run on paired-end reads and was initially used for read preprocessing. Raw reads were trimmed with Trimmomatic (v0.38) (52) using default parameters, followed by FastQC (v0.11.9) (https://www.bioinformatics.babraham.ac.uk/projects/fastqc/) for quality assessment. Trimmed reads were aligned to the *C. parapsilosis* CDC317 reference genome (version s01-m03-r49, CGD (53) using bwa mem (v0.7.17) (https://bio-bwa.sourceforge.net/bwa.shtml). Alignments were processed with samtools (v1.9) (54), and duplicate reads were marked with GATK MarkDuplicatesSpark (v4.1.2.0).

Small variants (single-nucleotide polymorphisms [SNPs] and insertions/deletions [INDELs]) were called assuming diploidy (--ploidy 2) with three independent callers: freebayes (v1.3.1), GATK HaplotypeCaller (v4.1.2.0) (55), and bcftools call (v1.9).

Structural variants (SVs) were identified using two complementary approaches:

1. Copy number variants (CNVs): deletions and duplications were inferred from read-depth changes across 300-bp windows using AneuFinder (v1.18.0) (56) and HMMcopy (v1.32.0) (57). CNVs spanning ≥600 bp (≥2 windows) were retained. Although some CNVs were initially detected, manual inspection in Integrative Genomics Viewer (IGV) (58) and read-depth visualization indicated that most arose from noisy coverage; therefore, CNVs were not further analyzed. Instead, we relied on per-gene coverage values obtained with mosdepth (v0.2.6) (59).
2. Breakpoint-resolved SVs: inversions, translocations, insertions, deletions, and tandem duplications were called based on split-read and discordant read-pair evidence, combined with local assembly. We used gridss (v2.9.2) (60) and clove (v0.17) (61). This method identified SV breakpoints. All SVs reported in this study refer to these breakpoint-defined variants.

Finally, both small variants and SVs were annotated using the Ensembl Variant Effect Predictor (VEP, v100.2) (62).

In summary, perSVade was used to generate filtered and annotated sets of SNPs, INDELs, and SVs, along with per-gene coverage estimates.

### Variant filtering and integration

To combine small variants, gene coverage measurements, and functional annotations, we used perSVade (see the supplemental material for details). Filtering was implemented using Python (v3.10.6) (63) and pandas (v1.5.1) (64).

For SV integration, we used perSVade (see the supplemental material for details). To refine the SV calls, we applied additional high-confidence filters, following the strategy in reference 65. Specifically, we calculated variant allele frequency (VAF) for each breakend and excluded variants with low VAF that may represent spurious rearrangements.

All SV filtering and integration steps were performed using Python and pandas.

### Comparison of variants across isolates

**Fig 1:**
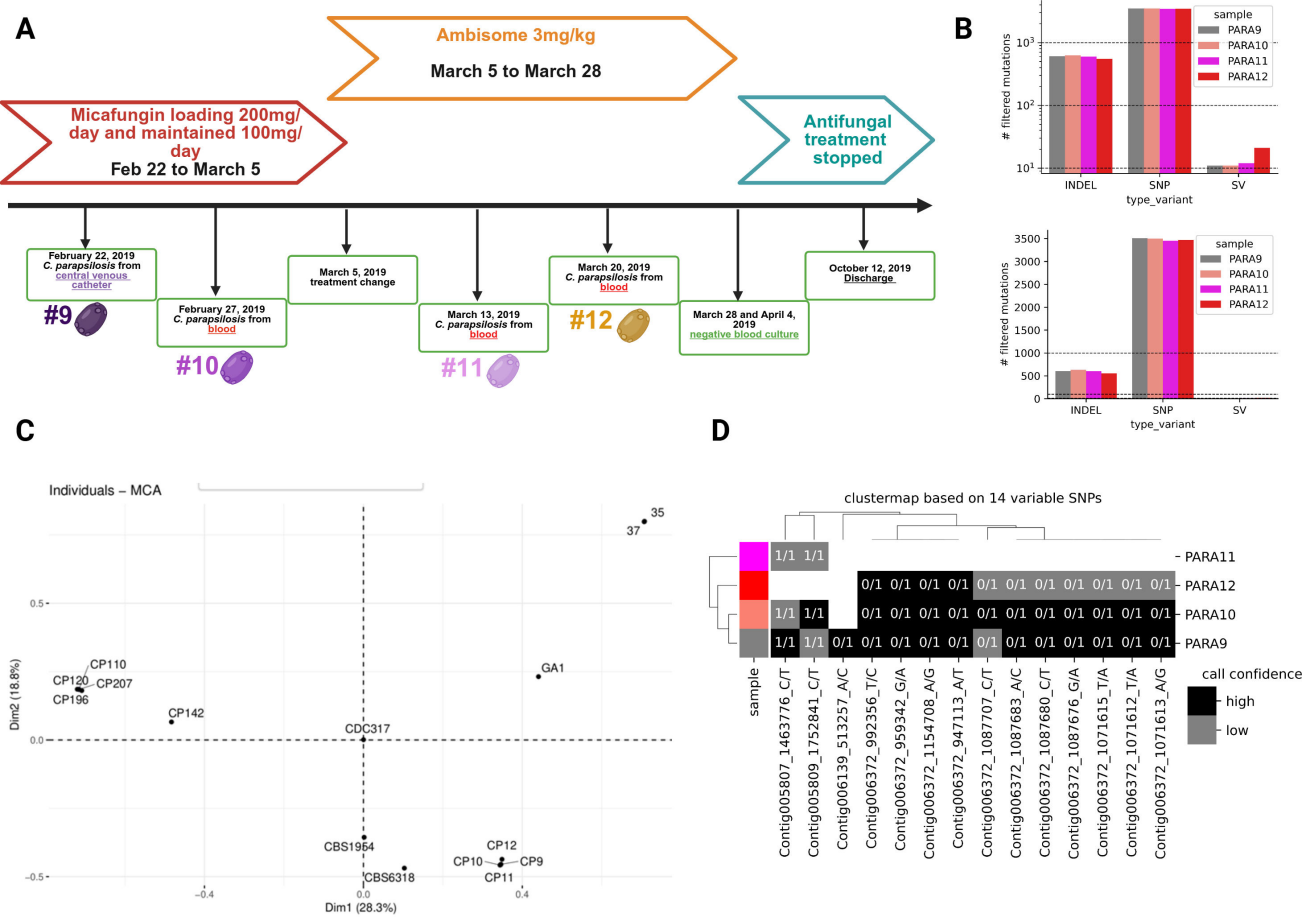
The clinical history and the WGS analysis of the serial *C. parapsilosis* isolates. Schematic diagram depicting the history of the patient suffering from persistent candidemia due to *C. parapsilosis* (note that Ambisome is the commercial name for liposomal AMB) (**A**). The number of variants identified in each isolate, in either linear (bottom) or log (top) scale (**B**). SNP-based multiple correspondence analysis (MCA) plot. Serial isolates formed a tight cluster with significant genetic distance relative to *C. parapsilosis* blood isolates collected from the same hospital (CP110, CP120, CP196, and CP207) (12) (**C**). Evolutionary relationships between isolates based on SNP data. Each column is one of the 14 SNPs that had a different presence/absence pattern across isolates (rows). The row colors match those shown in panel B. Clustermap generated using the function seaborn.clustermap, using “jaccard” as a distance metric and “average” as a linkage method for clustering the rows (**D**). The white, gray, and black colors represent SNPs not called, SNPs with low confidence not passing the quality control, and SNPs with high confidence passing the quality control, respectively. The clustering of SNPs and strains is based on the pattern of SNPs. 0/1 indicates heterozygous calls, while 1/1 indicates homozygous calls.

A key consideration in our variant analysis was how to compare variant sets between isolates. Although the isolates were clonal, they also showed divergence from the reference genome (Fig. 1B through D), necessitating a tailored approach for cross-isolate comparisons. Variant calling can introduce errors (false positives), so for each isolate, we applied stringent quality filters (see “Variant filtering and integration” in the supplemental material for details) to generate a “high confidence” set of variants. Only these variants were considered true for downstream analyses. However, because filtering itself can exclude genuine variants (false negatives), this creates challenges for comparisons across isolates.

To minimize false positives, we applied a custom approach to identify variants gained or lost in a given target isolate relative to a set of background isolates (Tables S3 and S6) (see the supplemental material for details). All filtering was performed using Python and pandas. This approach was used to identify variants present in isolates Cp10–Cp12 (targets) compared with the likely parental isolate Cp9 (background) (see Results; Tables S3 and S6; Fig. 1E). For variants shared among Cp10–Cp12 (Tables S4 and S5), we manually inspected alignments in IGV.

For broader presence/absence patterns across isolates (Table S2; Fig. 1D), we retained only variants that were high confidence as the identifier for SVs (see the supplemental material for details).

### MCA

To assess the clonality of isolates (Cp9-12), we performed an MCA that included our isolates and additional strains with previously published WGS data. These included clinical isolates CP110, CP120, CP142, CP196, and CP207 (corresponding to strains ECR, MDR1, MDR2, MDR3, and MDR4 from reference 12), as well as three unrelated strains of both clinical and environmental origin (GA1, CBS1954, and CBS6318) sequenced in reference 66. We also incorporated two *C. parapsilosis* clinical isolates (isolates 35 and 37), which were identified as part of a clonal cluster in a recent study (30), to serve as additional controls.

Raw sequencing data for all strains were processed with perSVade (v1.06) as described in “Variant calling and filtering.” SNPs were excluded if they had a mean mapping quality <30, QUAL <20, or read depth <30. Monomorphic SNPs were also removed. We retained only high-confidence variants, defined as those supported by at least two of perSVade’s variant callers, and generated a matrix of allele pairs for each SNP position across all isolates. The reference genome strain CDC317 was included as a baseline, contributing no SNPs.

MCA was then conducted in R using the dudi.acm function from the ade4 package (67), and results were visualized with fviz_mca_ind from the factoextra package (v1.0.5) (https://cran.r-project.org/web/packages/factoextra/index.html).

### Data visualization and statistical analysis

Plots related to genomic analyses were generated using seaborn (v0.12.1) (https://seaborn.pydata.org/), matplotlib (v3.6.2) (https://matplotlib.org/), and matplotlib-venn (v0.11.7) (https://github.com/konstantint/matplotlib-venn). GO enrichment analyses were carried out with goatools (v1.2.3) following the approach described in 65. Enrichment was initialized with the function GOEnrichmentStudyNS from goatools.goea.go_enrichment_ns using as input:

(i) the .obo ontology file (downloaded 01/31/2022 from https://current.geneontology.org/ontology/go-basic.obo), (ii) the GO terms assigned as described in “Functional annotations”, (iii) propagate_counts=True, (iv) all genes with at least one GO annotation, and (v) Benjamini–Hochberg FDR correction with a significance cutoff of *P* < 0.05 (methods=["fdr_bh"]).

The resulting GOEnrichmentStudyNS object was then analyzed with the function run study for each target gene set, defined either as (a) all genes containing private variants or (b) the subset of those variants predicted to alter protein sequences or transcripts. Enrichment was evaluated across all three GO namespaces: Biological Process, Molecular Function, and Cellular Component.

## RESULTS

### Evolution of serial *C. parapsilosis* isolates during infection

We analyzed 624 *C*. *parapsilosis* isolates collected from 532 candidemia patients across Turkey between 2009 and 2023 (10, 11, 31). To identify fungal evolution during active human infection, we applied strict criteria: patients had to be immunocompetent, exhibit persistent candidemia despite antifungal therapy, and yield serial isolates that remained phenotypically susceptible without known resistance mutations. Only one case met these criteria. The first isolate (Cp9) was recovered from a central venous catheter. Despite 12 days of micafungin therapy, candidemia persisted, and a second isolate (Cp10) was obtained (Fig. 1A). Switching to liposomal amphotericin B (LAMB) for ~3 weeks cleared the infection, during which two additional isolates (Cp11 and Cp12) were collected. All isolates remained susceptible to echinocandins and AMB by broth microdilution testing (Table S1).

To assess relatedness and identify potential resistance-associated mutations, we performed high-coverage (>100× ) paired-end WGS. Relative to the CDC317 reference genome, each isolate harbored ~3,500 SNPs, ~500 small INDELs, and 10–20 SVs including copy-number changes, deletions, duplications, inversions, and translocations (Fig. 1B). Variant loads were similar across isolates, indicating comparable genetic distances to the reference strain.

Direct comparison of the four clinical isolates identified only 14 unique SNPs, 42 INDELs, and 5 SVs (Table S2), consistent with clonality. Population structure analysis further supported a shared origin (Fig. 1C). Although limited genetic divergence constrained phylogenetic resolution, clustering revealed that 11/14 non-shared SNPs were heterozygous within a defined region on Contig006372 and absent in Cp11, consistent with a loss-of-heterozygosity event (Fig. 1D). The remaining three SNPs were unique to Cp9, supporting its role as the parental isolate.

Because micafungin therapy failed clinically, we examined variants within canonical antifungal resistance loci. No nonsynonymous mutations were detected in *ERG11*, *ERG3*, *TAC1*, *MRR1*, and *UPC2* (azoles); *FKS1* and *FKS2* (echinocandins); or *ERG2*, *ERG5*, *ERG6*, and *ERG25* (polyenes) (Table S2). Two upstream variants were detected: a structural change near *UPC2* in Cp12 and an INDEL upstream of *ERG2* in Cp10. Although these may influence expression, neither is a known resistance mechanism. Consistently, all isolates remained susceptible to azoles, echinocandins, and AMB.

These data show that the four sequential isolates were clonal and genetically stable at known antifungal resistance loci. Micafungin treatment failure therefore likely reflects microevolutionary changes that altered fungal tolerance rather than the acquisition of resistance, whereas LAMB remained effective in clearing the infection.

### *In vitro* growth analysis suggests CWR

To assess phenotypic differences among the serial isolates, we compared growth across diverse stress conditions. Growth rates were measured at 37°C in rich medium (YPD) and in YPD supplemented to impose low pH (pH 5), oxidative stress (10 mM H₂O₂), cell wall stress (Congo Red), membrane stress (SDS), osmotic stress (NaCl), and YPD containing 0.2% dextrose. Optical density at 600 nm was recorded hourly for 24 h, and final OD₆₀₀ values were used for statistical analysis.

**Fig 2:**
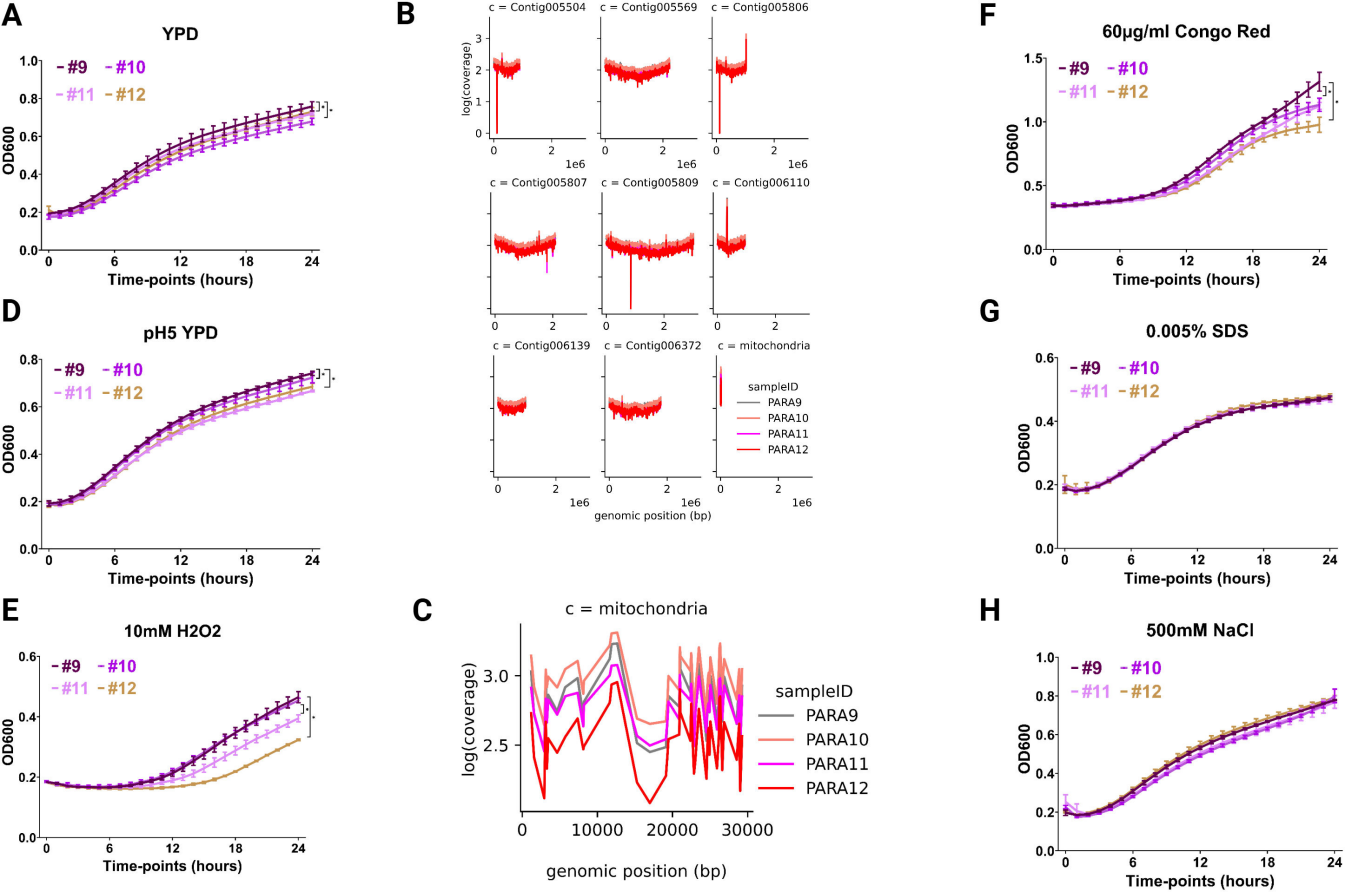
*C. parapsilosis* isolates collected later during the infection show *in vitro* growth defects. Growth curves of overnight cultures adjusted to a starting OD_600_ = 0.2 and resuspended in the indicated medium with OD_600_ values recorded hourly for 24 h are shown in panels **A**, **D**, **E**, **F**, **G**, and **H**. Conditions tested included rich YPD medium in the absence of any stress (**A**), pH = 5 YPD (**D**), oxidative stress, 10 mM H_2_O_2_ (**E**), cell wall stress, 60 µg/mL Congo Red (**F**), membrane stress, 0.005% SDS (**G**), and osmotic stress, 0.5M NaCl (**H**). Absolute coverage per gene on a log scale is shown along the genome (**B**) and along the mitochondrial genome (**C**). Differences in final OD_600_ values were used in statistical analysis and * denotes *P* ≤ 0.05. The Wilcoxon signed-rank test of the final OD_600_ values was used for comparative analysis.

In stress-free YPD, Cp9 exhibited the fastest growth, whereas Cp12 grew slowest (Fig. 2A). Because reduced growth in rich medium can indicate mitochondrial defects, we also assessed colony morphology and mitochondrial DNA content. Both appeared comparable across isolates, ruling out mitochondrial loss as the cause (Fig. 2B and C). A similar growth pattern was observed at lower pH, where Cp9 also outperformed later isolates in the pH5 YPD condition (Fig. 2D).

Growth disparities were amplified under certain stress conditions. Cp9 consistently outperformed later isolates in conditions of oxidative stress (Fig. 2E) and cell wall stress (Fig. 2F). No notable differences were observed under membrane stress (Fig. 2G). Under osmotic stress, Cp12 grew similarly to Cp9, whereas Cp10 and Cp11 exhibited delayed early growth (Fig. 2H).

These findings demonstrate that the later isolates display reduced fitness under multiple stresses, most notably oxidative and cell wall stresses, suggesting that evolutionary adaptation within the host involved CWR at the expense of stress tolerance.

### Serial isolates undergo progressive CWR

**Fig 3:**
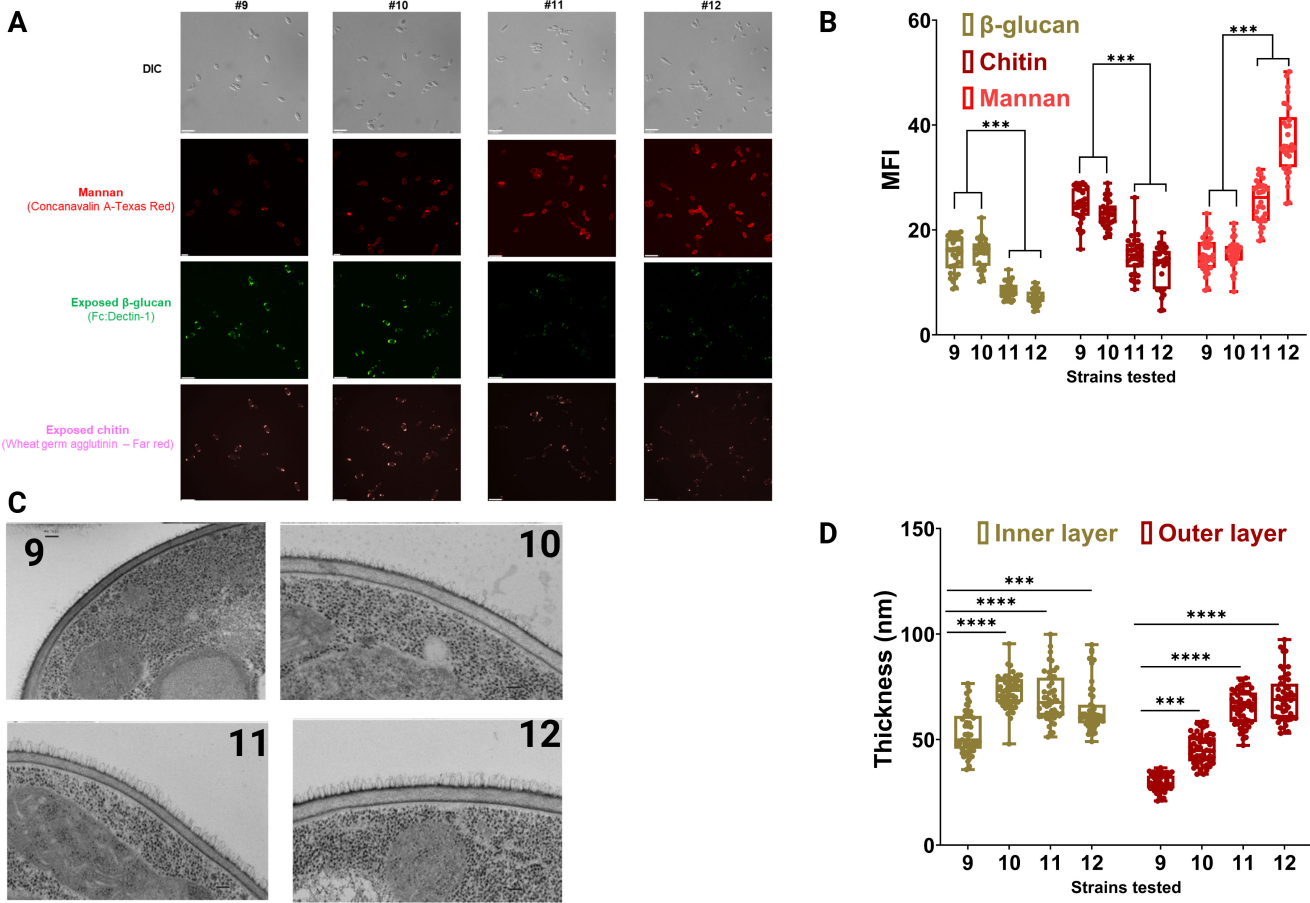
*C. parapsilosis* isolates underwent CWR during infection. Cell wall exposure analysis of *C. parapsilosis* isolates using fluorescence microscopy (**A**) and pertinent MFI values (**B**). Chitin, β-glucan, and mannan exposures were determined using wheat germ agglutinin, anti–dectin-1 antibody, and concanavalin A. TEM graphs of *C. parapsilosis* isolates (**C**) and pertinent inner and outer cell wall thickness in nm (**D**). *** *P* ≤ 0.001 and *P* ≤ 0.0001. The Wilcoxon Signed Ranks Test was used for analysis.

The reduced fitness of isolates of Cp10–Cp12 under cell wall stress (Fig. 2F) prompted a detailed investigation of their cell wall architecture. We first examined surface carbohydrate exposure by fluorescence microscopy using wheat germ agglutinin (chitin), anti–dectin-1 antibody (β-glucan), and concanavalin A (mannan). Across the isolate series, we observed a progressive decrease in exposed chitin and β-glucan accompanied by increased mannan signals, with the most pronounced changes in Cp11 and Cp12 (Fig. 3A and B). HPF-TEM corroborated these findings, revealing thickening of both the fibrillar mannan layer and the inner wall structure in later isolates (Fig. 3C and D). Together, these results indicate substantial CWR during infection, consistent with adaptive responses reported in *C. albicans* under echinocandin stress (68).

**Fig 4:**
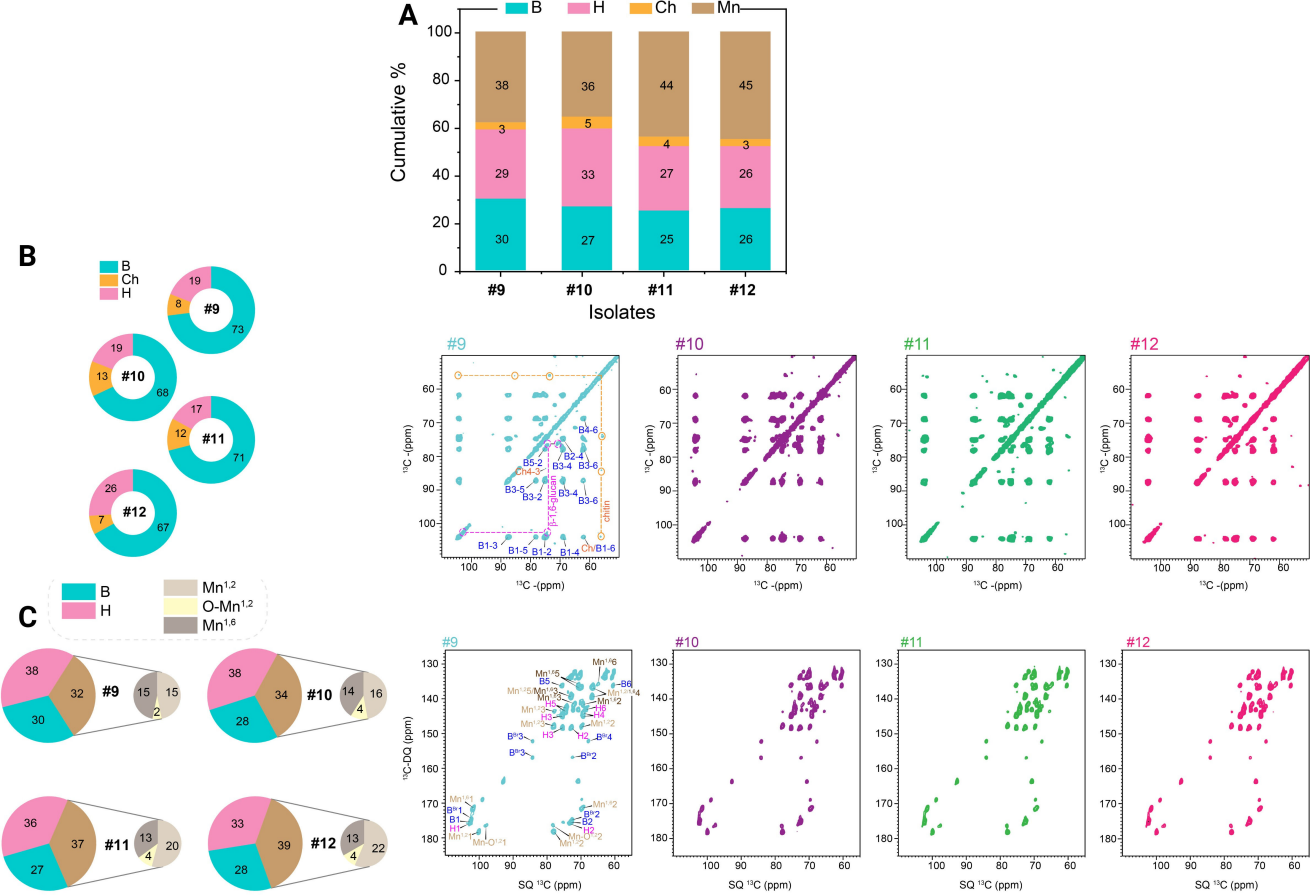
Determination of the chemical composition of the cell wall structure using solid-state NMR. Overall cell wall composition (rigid and mobile sections) using solid-state NMR (**A**). The rigid fraction of the cell wall (**B**). 2D CP-based ^13^C-^13^C CORD spectra of Cp9, Cp10, Cp11, and Cp12, shown in turquoise, purple, green, and pink, respectively. Cross-peak assignments for the resolved polysaccharides are labeled with glucans color-coded as follows: β−1,3-glucans (blue), chitin (orange) (also indicated by orange dashed lines and circles highlighting chitin cross-peaks), and β−1,6-glucans (magenta), with corresponding magenta lines and circles. All isolates exhibit similar glucan components, with variations in cross-peak intensities (**B**). The mobile fraction and mannan composition (**C**). 2D DP *J*-INADEQUATE spectra highlighting the mobile components in Cp9, Cp10, Cp11, and Cp12 are shown in turquoise, purple, green, and pink, respectively. Through-bond carbon correlations for the resolved polysaccharides are labeled in Cp9, with glucans and mannans color-coded as follows: β−1,3-glucans (blue), β−1,6-glucans (magenta), and mannans (various shades of brown)-α−1,2-mannans (brown, Mn^1,2^), oxygen-linked α−1,2-mannans (light brown, O-Mn^1,2^), and α−1,6-mannans (gray, Mn^1,6^). While all isolates share similar glucan components, differences in peak intensities are observed (**C**). All spectral processing and peak integrations were performed using TopSpin 4.1.4, calculations for relative abundance were conducted in Excel, and graphical representations were generated using Origin 2021.

To quantify cell wall composition, we performed solid-state NMR spectroscopy on intact cells (69, 70). While core polysaccharide classes were conserved, we detected marked compositional shifts. Relative to Cp9, Cp11 and Cp12 exhibited higher mannan content (44%–45% vs 38%) and reduced β−1,3/β−1,6-glucan (52% vs 59%), whereas Cp10 most closely resembled Cp9 (Fig. 4A). Chitin levels (3%–5%) were modestly elevated in Cp10 but did not substantially change in later isolates. Thus, unlike *C. albicans* (16), which frequently increases chitin to compensate for glucan loss, *C. parapsilosis* appears to rely primarily on mannan expansion.

We next resolved rigid (chitin and β-glucans) versus mobile (mannans and glucans) wall fractions. In the rigid fraction, Cp10–Cp11 showed reduced β−1,3-glucan and increased chitin, whereas Cp12 exhibited the highest β−1,6-glucan levels (Fig. 4B). In the mobile fraction, all later isolates displayed progressive reductions in β−1,3-glucan, Cp11–Cp12 showed decreased β−1,6-glucan, and Cp10–Cp12 demonstrated steadily increasing mannan levels (Fig. 4C). Mannan sub-classification revealed subtle but consistent increases in O-linked α−1,2-mannan and branched N-linked mannan (α−1,2 and α−1,6), suggesting enhanced cell surface flexibility and altered host interactions.

These analyses demonstrate extensive *in vivo* CWR across serial *C. parapsilosis* isolates. The glucan–chitin scaffold was maintained, but mannan abundance and branching progressively increased while β-glucan exposure decreased. These adaptations likely influence stress resilience and could potentially modulate immune detection during persistent infection.

### CWR promotes thicker, denser biofilms

*C. parapsilosis* is well known for forming robust biofilms that support persistence on host and abiotic surfaces and complicate antifungal treatment (71). The biofilm extracellular matrix (ECM) in this species is enriched in mannans, with additional contributions from β-1,6- and β-1,3-glucans (71–74). Given the increased mannan content in Cp11–Cp12 and elevated β-1,6-glucan in Cp10, we hypothesized that later isolates would exhibit enhanced biofilm formation, potentially contributing to echinocandin tolerance in the absence of canonical *FKS* mutations.

**Fig 5:**
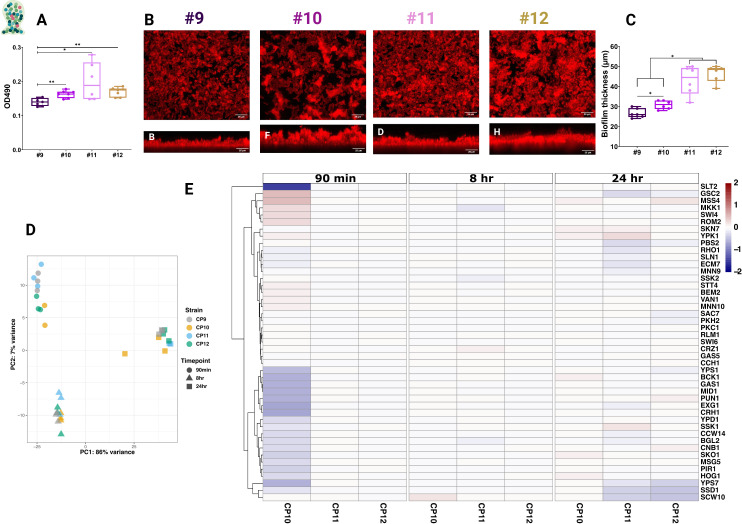
Determination of the biofilm level of *C. parapsilosis* isolates under *in vitro* conditions and expression of cell wall-related genes at various stages of biofilm formation. Determination of biofilm level of *C. parapsilosis* isolates using crystal violet (**A**) and CLSM (**B and C**). Principal component analysis (PCA) plot of *C. parapsilosis* isolates under planktonic conditions (**D**). Variance stabilizing transformation was applied to planktonic samples for the PCA plot. The x-axis shows the dimension with most variance computed using the top 500 genes that have the most variance across samples, and the y-axis shows PC2, with the second-most variation in data. Expression of cell-wall related genes in planktonic condition across strains Cp10–Cp12 relative to Cp9 at each time point (**E**). * *P* ≤ 0.05 and ** *P* ≤ 0.01. The Wilcoxon Signed Rank Test was used for analysis.

Quantitative crystal violet assays demonstrated that Cp10–Cp12 produced significantly more biofilm biomass than the initial isolate Cp9 (Fig. 5A). CSLM further showed that biofilms formed by Cp11 and Cp12 were markedly thicker and more compact than those produced by Cp9 and Cp10 (Fig. 5B and C), consistent with increased ECM deposition.

These findings suggest that in-host evolution of *C. parapsilosis* is accompanied by biofilm augmentation, likely driven in part by increased mannan and β-glucan remodeling. This enhanced biofilm architecture may contribute to the heightened resilience of later isolates under antifungal pressure and host immune challenge.

### Comparative genomics indicates nonconvergent microevolution

To investigate the genetic basis of the observed cell wall and biofilm phenotypes, we compared genomes of the evolved isolates (Cp10–Cp12) to the initial isolate (Cp9). Across the three later isolates, we identified 50 isolate-specific small variants (SNPs/INDELs) and four SVs affecting 208 genes (Table S3). Variant sets were largely non-overlapping (22 in Cp10, 26 in Cp11, and 19 in Cp12; Fig. S1A), suggesting independent microevolutionary paths rather than a single dominant adaptive trajectory.

Only 10 of the 208 affected genes (4.8%) carried variants predicted to affect protein function, and no significant GO enrichments were detected, consistent with scattered low-impact variation. Among the few variants of interest, an in-frame deletion in *CPAR2_208870* (encoding a serine/threonine kinase ortholog involved in G2/M regulation [75]) was present in Cp10 and Cp12, while other changes were isolate-specific, including variants in *MUB1* and two adhesin genes (*CPAR2_404790* and *CPAR2_404800*). Aside from these adhesin genes, none overlapped with loci implicated in CWR in *Candida auris* clinical isolates (20, 21).

To identify potential shared adaptive events, we searched for variants present in Cp9 but lost in Cp10–Cp12. Four such heterozygous noncoding variants (3 INDELs and 1 SNP) were validated in IGV (Tables S4 and S5), mapping near 16 genes enriched for metabolic regulators. While these loci did not show strong adaptive signatures in *C. parapsilosis*, orthologs of four (*ENA2, RFC3, CPAR2_211420,* and *CPAR2_502250*) displayed elevated selection scores in other *Candida* species (65), suggesting potential evolutionary relevance.

These results indicate that these *C. parapsilosis* serial isolates evolved through complex, largely isolate-specific microevolution during persistent infection, without clear convergence at known antifungal or cell wall-regulatory loci. The phenotypic shifts in cell wall architecture and biofilm formation therefore likely reflect polygenic and/or regulatory adaptation rather than acquisition of classical resistance mutations.

### Transcriptomic analysis links CWR and biofilm phenotypes to stage-specific responses

To investigate the molecular basis of CWR and enhanced biofilm formation, we compared transcriptomes of Cp9–Cp12 under planktonic and biofilm conditions at 90 min, 8 h, and 24 h, corresponding to adhesion, initiation, and maturation stages of biofilm development.

Under planktonic conditions, Cp10–Cp12 displayed broadly similar transcriptional profiles relative to Cp9 across all time points (Fig. 5D). GO enrichment revealed few differences, with downregulation of “fungal-type cell wall” genes in Cp11 and Cp12 at 24 h (Fig S1B through E). Differentially expressed genes included *CPAR2_300120* (*C. albicans CSA1* ortholog), *CPAR2_403510* (*C. albicans RBT1* ortholog), and *CPAR2_806670* (*C. albicans YWP1* ortholog), the latter encoding a secreted cell wall protein that regulates β-glucan exposure and biofilm thickness in *C. albicans* (76–79). Broader analysis of cell wall–associated transcripts highlighted downregulation of *YPS7* (encoding a GPI-anchored aspartyl protease involved in adhesin release), *SSD1* (encoding a cell wall integrity regulator), and *SCW10* (encoding a glucanase/transglucosidase) in Cp11–Cp12 at 24 h (Fig. 5E; Table S7). These coordinated changes are consistent with the pronounced CWR observed in later isolates. Notably, Ssd1 regulates cell wall integrity, and its deletion causes cell wall defects and resistance to fludioxonil in *S. cerevisiae* (80, 81), while *scw4Δ/scw10Δ* double-mutant strains exhibit elevated chitin and mannan in *S. cerevisiae* (82), and Yps7 is required for adhesin processing and adherence in *C. glabrata* (83).

**Fig 6:**
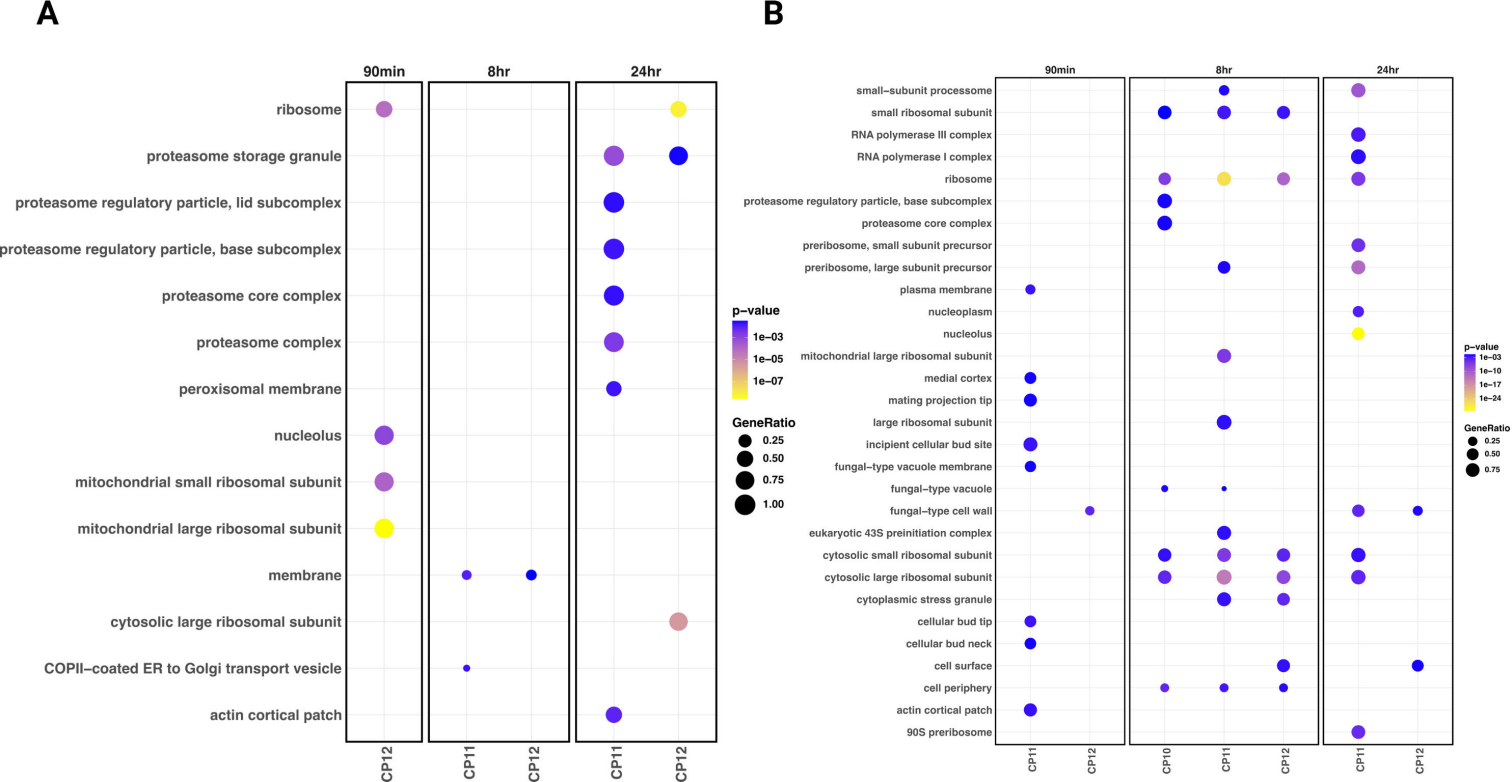
Transcriptomic responses of *C. parapsilosis* isolates at various stages of biofilm formation. Cellular components (**A**) upregulated and (**B**) downregulated in Cp10–Cp12 relative to Cp9 at each time point in the biofilm condition. Log2 fold change of gene expression in Cp10–Cp12 was obtained for each strain relative to Cp9 in each time point in the biofilm condition to identify strain-specific changes in expression. Figure depicts (**A**) upregulated and (**B**) downregulated cellular components that are enriched in at least one of the Cp10–Cp12 strains at a biofilm time point relative to Cp9.

Biofilm transcriptomes were distinct from planktonic states (Fig. S2B). PCA revealed minimal divergence among isolates (Fig. S2A), but stage-specific functional differences emerged. At 8 h, later isolates upregulated amino acid and transmembrane transport while downregulating translation, consistent with transient amino acid starvation during biofilm initiation (Fig. S2C and D; Fig. 6A). By 24 h, Cp11–Cp12 downregulated multiple genes encoding cell wall-modifying enzymes, including chitinase (*C. albicans CHT3* ortholog), β-glucosidase (*C. albicans SUN41* ortholog), and glucanase (*C. albicans SCW11* ortholog) (Fig. 6B; Table S8). These enzymes regulate cell separation (84), and their suppression may favor filamentation (85), consistent with the thicker biofilms of Cp11–Cp12. The coordinated downregulation of translation and upregulation of amino acid transport suggests metabolic adaptation to restore protein synthesis following nutrient limitation, reminiscent of regulatory responses observed in *C. albicans* amino acid permease mutants (86).

Comparative GO analyses across isolates and time points revealed modest strain-specific differences, with no processes uniquely associated with later isolates in biofilm formation or CWR (Fig. S3A and B). Temporal profiling of biofilm-associated and cell wall genes—including orthologs of known *C. albicans* biofilm regulators *CZF1, UME6, CPH2, GZF3, ACE2, BRG1, EFG1, GAL4,* and *NDT80* (87) —showed largely similar expression across isolates, except for downregulation of *UME6*, *BRG1*, *EFG1*, *GAL4*, and *NDT80* in Cp11 at 24 h (Fig. S4A and B; Table S8), suggesting potential species-specific regulatory differences in *C. parapsilosis*.

Results from the integration of our RNA-seq and WGS data are described in detail in the supplemental material.

Overall, transcriptomic profiling revealed largely conserved expression landscapes across isolates, with stage- and condition-specific differences in Cp11–Cp12. Downregulation of key cell wall–modifying enzymes and regulators, coupled with metabolic adaptations during biofilm initiation, likely underpins their enhanced CWR and ability to form thicker, more compact biofilms.

### Later isolates exhibit enhanced echinocandin tolerance but increased AMB susceptibility

The later isolates (Cp10–Cp12) underwent pronounced CWR, marked by reduced β-glucan and increased mannan, coinciding with thicker biofilms. Since β-glucan reduction is a known adaptive response to echinocandins, we hypothesized that micafungin treatment failure could reflect this CWR, despite all isolates remaining phenotypically susceptible and lacking *FKS* mutations beyond naturally occurring polymorphisms.

**Fig 7:**
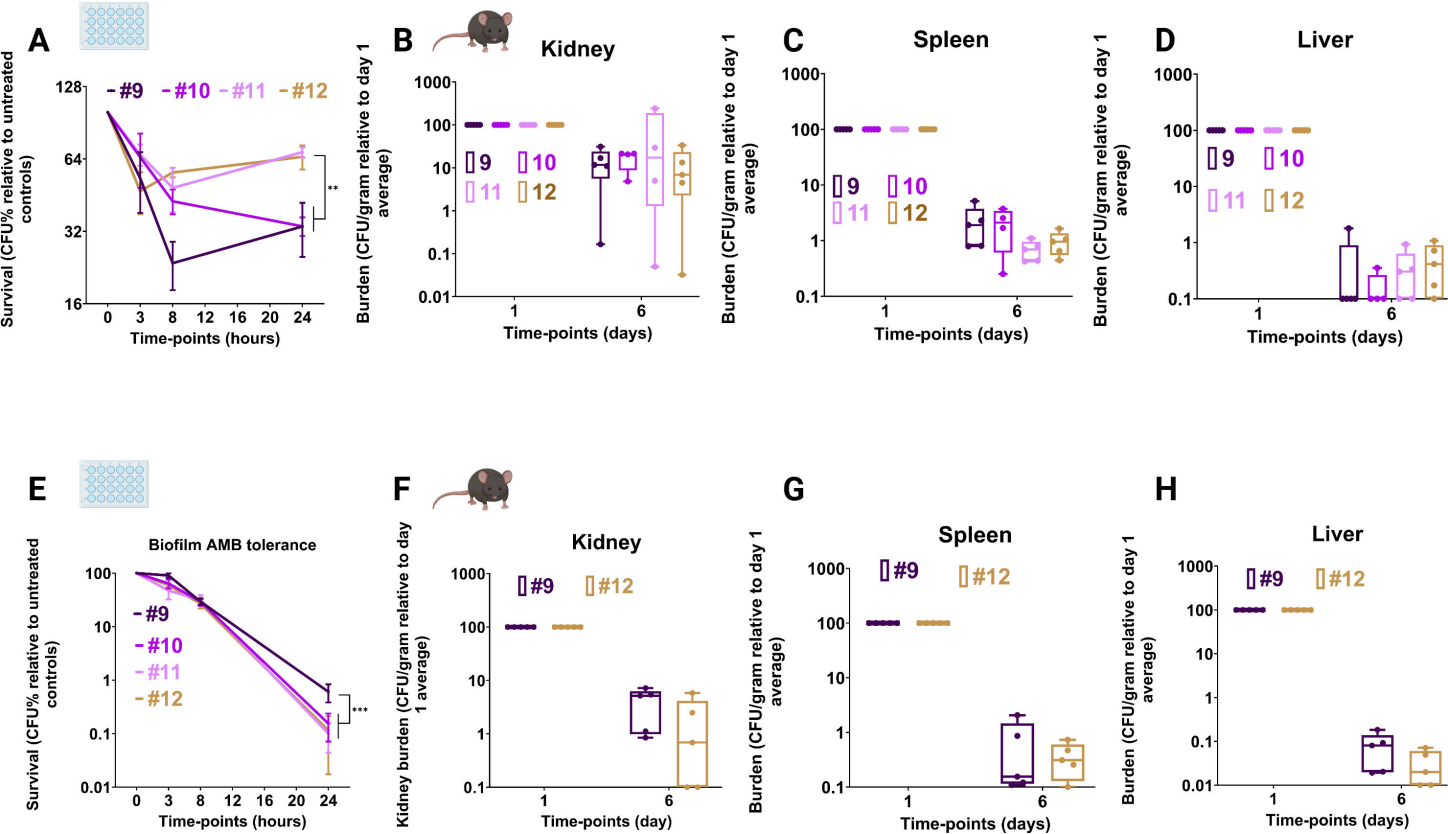
*In vitro* and *in vivo* determination of echinocandin and AMB tolerance of the *C. parapsilosis* isolates. Tolerance of biofilms of the serial isolates to micafungin *in vitro* (**A**). Tolerance of the serial isolates to caspofungin in a systemic infection mouse model of candidemia based on CFU enumeration in the kidney (**B**), spleen (**C**), and liver (**D**). Tolerance of biofilms of the serial isolates to AMB *in vitro* (**E**). Tolerance of the serial isolates to LAMB in a systemic infection mouse model of candidemia based on CFU enumerations in the kidney (**F**), spleen (**G**), and liver (**H**). *In vitro* antifungal biofilm tolerance determination used fully mature biofilms after 3-, 8-, and 24-h antifungal drug exposure by plating and CFU enumeration against untreated controls at pertinent time points. *In vivo* antifungal tolerance determination used C57BL/6 mice intravenously infected with 2 × 10^7^ fungal cells. Organs were harvested at designated time points, homogenized, and plated on YPD plates for CFU counting. Antifungal treatment (5 mg/kg) was started 24 h post-infection, and 5 mice were used for each isolate at each time point (each dot represents one mouse). ** *P* ≤ 0.01 and *** *P* ≤ 0.001. The Wilcoxon Signed Rank Test was used for analysis.

We focused on biofilms because they were positively selected during infection and provide protection against both host defenses and antimicrobial drugs (88). Mature 24-h biofilms were exposed to high micafungin concentrations (64 µg/mL; 64-fold above the MIC). After disruption with proteinase K, CFUs were normalized to untreated controls. Cp10–Cp12 showed increased survival relative to Cp9 at early time points, with Cp11 and Cp12 maintaining significantly higher survival at 24 h (Fig. 7A), indicating that evolved CWR likely confers enhanced echinocandin tolerance in biofilm contexts.

To validate these findings *in vivo*, immunocompetent mice were systemically infected and treated with humanized micafungin doses (5 mg/kg) (89). Fungal burdens in the kidney (Fig. 7B) and spleen (Fig. 7C) were comparable across isolates, likely reflecting lower echinocandin penetration in these organs. In the liver, a greater number of mice infected with Cp11 (*n* = 3) and Cp12 (*n* = 4) had detectable CFUs compared to Cp9 (*n* = 1) (Fig. 7D), consistent with a trend—albeit not statistically significant—toward increased tolerance where echinocandins penetrate more effectively (90–92).

Because lower β-glucan content can increase AMB susceptibility (93), we assessed mature biofilms with high-dose AMB (64 µg/mL). Cp10–Cp12 displayed significantly reduced survival relative to Cp9 (Fig. 7E), suggesting that CWR, while enhancing echinocandin tolerance, simultaneously increases AMB sensitivity. Systemic infection experiments with LAMB (5 mg/kg, 3 doses) (94) mirrored this trend, though differences did not reach statistical significance (Fig. 7F through H). These findings are reminiscent of the collateral sensitivity phenomena, where acquisition of resistance to one antifungal drug could increase susceptibility to another (95, 96).

These results demonstrate that CWR in later *C. parapsilosis* isolates produces a dual phenotype: enhanced echinocandin tolerance in biofilms but heightened vulnerability to AMB. This trade-off identified a potential “Achilles’ heel” and provides support for therapeutic strategies that alternate echinocandin and LAMB treatment to eradicate persistent *C. parapsilosis* infections.

### CWR enhances immune evasion and yields transient fitness gains *in vivo*

**Fig 8:**
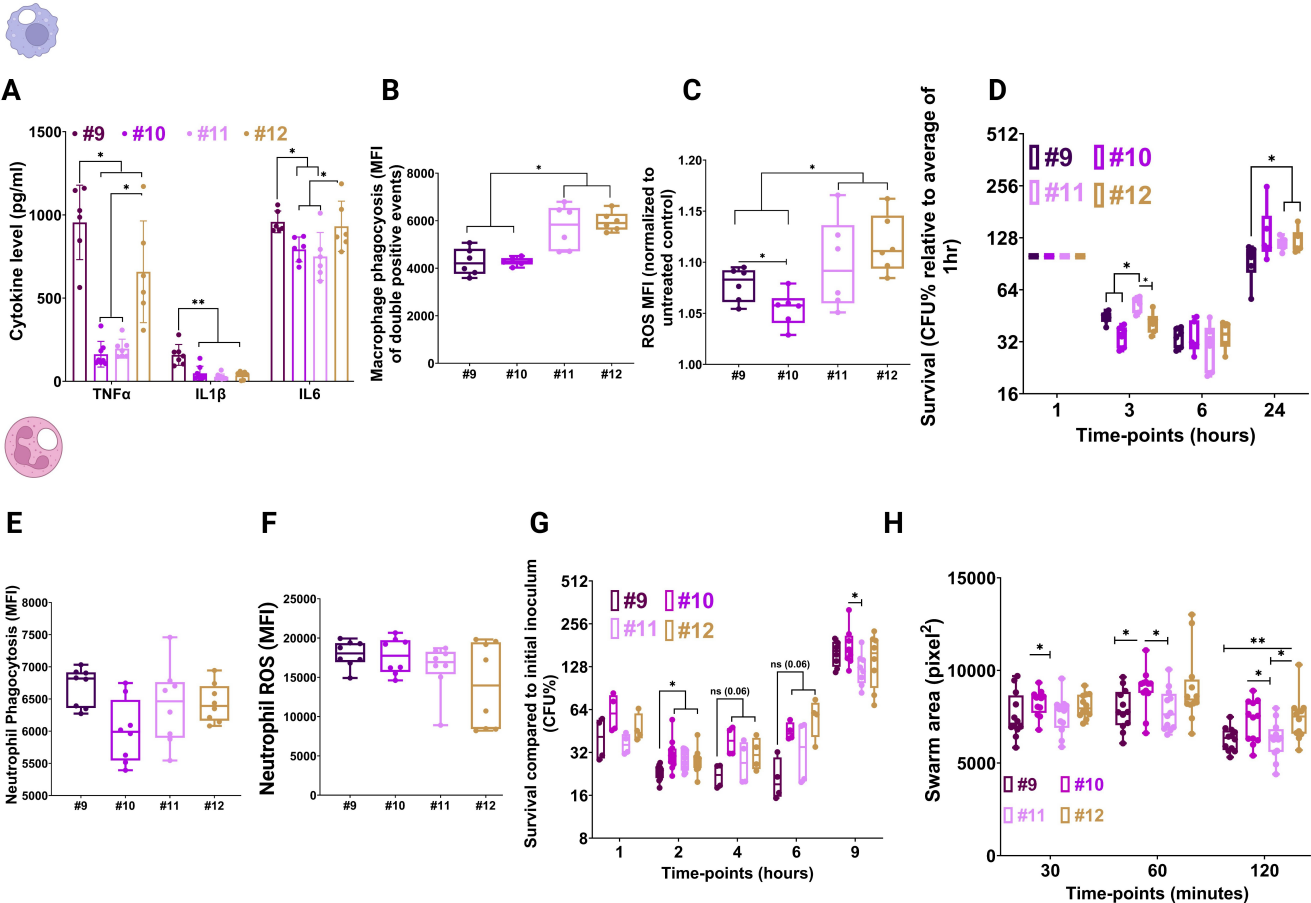
Interaction of *C. parapsilosis* isolates with primary human macrophages and neutrophils. Production of proinflammatory cytokines (**A**), phagocytosis (**B**), and ROS production (**C**) induced by the serial isolates during interaction with primary human macrophages. Survival during interaction with primary human macrophages (**D**). Phagocytosis (**E**) and ROS production (**F**) induced by serial isolates during interactions with primary human neutrophils. Survival during interaction with primary human neutrophils (**G**). Cytokine concentrations were measured by ELISA at 24 h post-infection, whereas phagocytosis was measured by MFI of FITC-labeled *C. parapsilosis* at 1 h post-infection (only intracellular cells), and macrophage/neutrophil ROS was determined by measuring the MFI of dihydrorhodamine 123 at 3 h post-infection. Survival during interaction with neutrophils was determined by normalizing CFUs at designated time points against the initial inoculum. Survival during interaction with macrophages was measured by normalizing intracellular CFUs at designated time points against pertinent intracellular cells at 1 h post-infection. Swarming of primary human neutrophils in the presence of the serial isolates (**H**). * *P* ≤ 0.05 and ** *P* ≤ 0.01. The Wilcoxon Signed Rank Test was used for analysis.

Human monocyte-derived macrophages (hMDMs) infected at an MOI of 10 exhibited significantly lower proinflammatory cytokine production (IL-1β, TNF-α, and IL-6) in response to Cp10–Cp12 compared to Cp9, with Cp11–Cp12 eliciting the greatest suppression (Fig. 8A). Reduced β-glucan exposure likely contributed to diminished dectin-1 engagement. Surprisingly, phagocytosis assays revealed that Cp11–Cp12 were more efficiently internalized than Cp9 and Cp10 (Fig. 8B), confirmed by CFU enumeration (Fig. S5). ROS production was elevated in Cp11–Cp12 infections, yet these isolates exhibited significantly higher intracellular survival at 24 h (Fig. 8D), indicating that adaptive CWR suppresses proinflammatory signaling without compromising, and in some cases enhancing, fungal fitness within macrophages.

Interactions with primary human neutrophils showed comparable phagocytosis and ROS induction across isolates (Fig. 8E and F), but Cp10–Cp12 maintained higher survival at early time points (2–6 h), and neutrophil swarming toward Cp10 and Cp12 was more pronounced at later stages (Fig. 8G and H).

**Fig 9:**
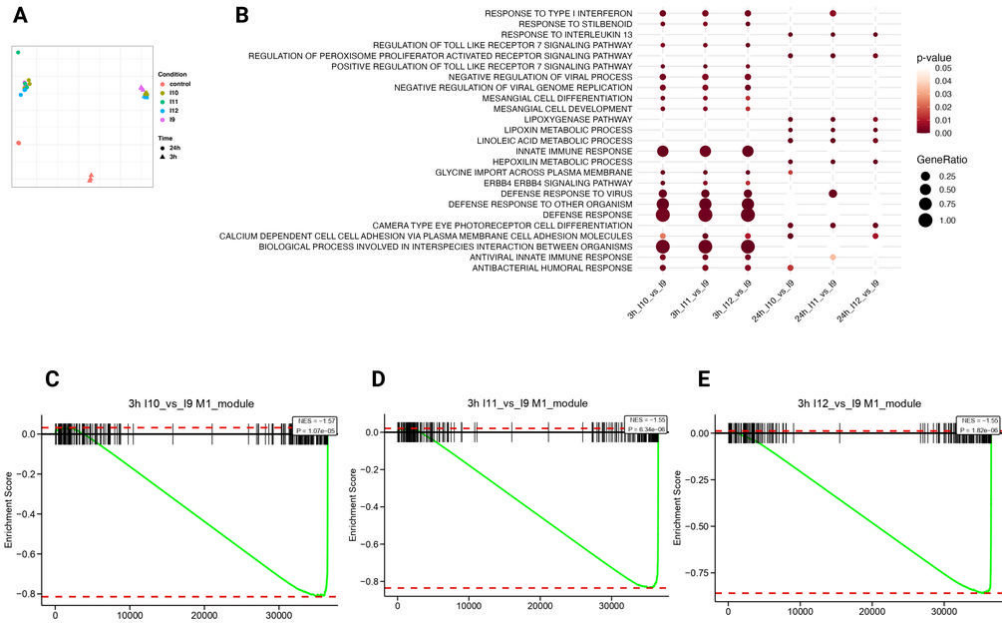
Transcriptomic responses of macrophages toward serial isolates of *C. parapsilosis*. PCA plot of infected macrophages and uninfected macrophages (**A**). GO-term pathway analysis (upregulated shown) of macrophages infected with the later isolates (**B**). Enrichment of transcripts associated with M1 polarized macrophages (marked as black vertical lines) infected in the later isolates estimated using GSEA (**C–E**). The x-axis shows the ranked order of the transcripts by fold change, and the y-axis shows the enrichment score, a running sum statistic based on the ranked order of genes. The negative NES and *P*-values indicate that transcripts associated with M1 polarized macrophages are significantly enriched among downregulated genes in later isolates.

To explore molecular mechanisms, RNA-seq was performed on hMDMs infected at MOI 5, with RNA collected at 3 and 24 h. PCA revealed robust infection-induced transcriptomic changes relative to uninfected controls, while differences among isolates were modest (Fig. 9A). At 3 h, GO analysis revealed selective upregulation of type I interferon and TLR7 signaling in Cp10–Cp12–infected macrophages (Fig. 9B). Importantly, M1-associated transcripts were suppressed in macrophages infected with Cp10–Cp12 relative to Cp9 (Fig. 9C through E), consistent with reduced cytokine production and enhanced intracellular survival. These data suggest that adaptive CWR modulates early macrophage polarization, promoting immune evasion while preserving fungal fitness.

**Fig 10:**
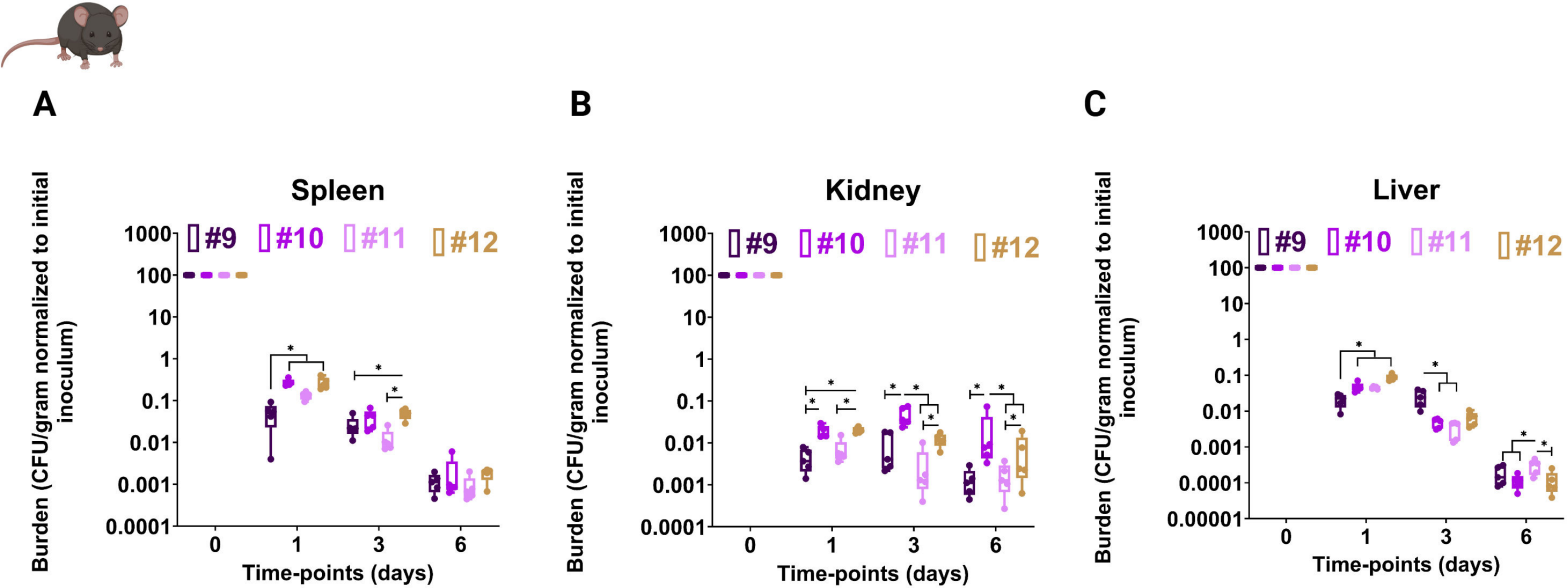
Later *C. parapsilosis* isolates had a transiently higher survival in a systemic infection mouse model of candidemia**.** CFUs at each timepoint are shown in the spleen (**A**), kidney (**B**), and liver (**C**). Each dot represents a single mouse. C57BL/6 mice intravenously infected with 2 × 10^7^ fungal cells, organs were harvested at designated time points, homogenized, and plated on YPD plates for CFU counting. Five mice were used for each isolate at each time point. * *P* ≤ 0.05. The Wilcoxon signed-rank test was used for analysis.

To determine whether these *in vitro* advantages translate *in vivo*, mice were systemically infected via tail vein with Cp9–Cp12, and fungal burdens were measured in kidney, liver, and spleen over time. Neutrophil recruitment peaks around 24 h in this model with *C. albicans* (97). On day 1, Cp10–Cp12 exhibited significantly higher fungal burdens across all organs (Fig. 10A through C), reflecting enhanced early survival consistent with efficient neutrophil interactions observed *in vitro*. Organ-specific dynamics emerged over time: in the spleen, Cp12-infected mice maintained higher burdens at day 3; in the kidney, Cp10 and Cp12 consistently displayed elevated loads; and in the liver, Cp9 peaked at day 3, Cp11 at day 6, while Cp10 and Cp12 showed intermediate burdens (Fig. 10A through C).

In summary, these findings demonstrate that adaptive CWR and echinocandin tolerance in later isolates could suppress host proinflammatory responses, modulate macrophage polarization, and confer transient, organ- and timepoint-specific fitness advantages during systemic infection. Overall, these results highlight the context-dependent benefits of CWR in maintaining fungal survival and persistence within the host.

## DISCUSSION

*C. parapsilosis* represents a significant public health threat, with rising reports of FLCR, ECR, and MDR isolates (1). Understanding how this species persists in the host and withstands antifungal treatment remains essential. Although antifungal tolerance is often linked to reduced virulence (23, 25, 98), whether fungal pathogens can concurrently resist innate immune defenses and tolerate antifungal drugs—and what cellular adaptations enable this—is unclear.

Here, using a longitudinal series of bloodstream isolates from a patient with persistent *C. parapsilosis* candidemia, we show that this species can maintain virulence during persistent infection while progressively acquiring echinocandin tolerance. These later isolates exhibited extensive CWR, markedly increased mannan and reduced β-glucan, and enhanced biofilm formation. Despite lacking *FKS1* mutations beyond the intrinsic P660A variant (99), these isolates displayed increased micafungin tolerance, consistent with the hypothesis that subtherapeutic echinocandin exposure or tissue-specific drug penetration can drive adaptive responses (90–92).

A core finding is that adaptive CWR in *C. parapsilosis* diverges from canonical chitin-upregulation pathways described in other *Candida* species (100–103). Instead, the later isolates increased O- and N-linked mannan without detectable increases in chitin. RNA-seq did not support activation of classic CWR pathways, and WGS failed to identify shared mutations across later isolates, suggesting polygenic and multifactorial adaptations under combined host and drug pressures. Integration of RNA-seq and WGS data further indicated that the pronounced CWR and biofilm phenotypes in the later serial isolates (Cp11–Cp12) likely result from a complex interplay of subtle transcriptomic and polygenic changes rather than single canonical mutations. These results highlight that the molecular determinants of mannan remodeling—and its contribution to echinocandin tolerance and biofilm architecture—are poorly defined and warrant future investigation.

CWR had profound consequences for immune interactions. Despite pronounced β-glucan masking, later isolates were more efficiently phagocytosed by macrophages, yet triggered lower pro-inflammatory cytokine production and suppressed early M1 polarization. This contrasts with classical models in *C. albicans* where reduced mannan exposure enhances immune activation (104, 105). The elevated mannan levels in our isolates likely modulate immunity through alternative mechanisms: mannose-receptor engagement can dampen NF-κB activation (106), suppress glycolysis-driven inflammatory pathways (107), and reduce cytokine output, consistent with our transcriptomic and cytokine measurements. We propose that mannan degradation within phagosomes may release mannose ligands that skew macrophage metabolism and polarization toward a less inflammatory state.

Notably, phagocytosis and ROS production diverged. While later isolates were phagocytosed more efficiently, ROS responses remained similar to the initial isolate, and neutrophil-induced ROS was unchanged. These results emphasize that β-glucan-centric paradigms—particularly dectin-1-driven recognition (104, 108, 109)—do not fully explain immune sensing in *C. parapsilosis*. Instead, the highly variable mannan layer (110, 111) likely plays a dominant role, and our data argue for a broader view of fungal recognition pathways.

Adaptation to echinocandins was accompanied by a clinically important trade-off: increased susceptibility to AMB. Consistent with prior work (93, 95), reduced β-glucan content heightened AMB vulnerability, and clinical resolution coincided with LAMB therapy. This antimicrobial “Achilles' heel” suggests that strategic alternation between echinocandins and AMB—or timed sequential therapy—may exploit vulnerabilities of echinocandin-adapted isolates.

Our study has limitations. We analyzed a single colony per clinical time point from a single patient, which likely underestimates intra-host fungal diversity and could overlook parallel adaptive trajectories. Additionally, the multifactorial genetic and transcriptional changes observed across later isolates complicate definitive mechanistic assignment and suggest that adaptation proceeds via polygenic and context-dependent routes rather than a single dominant pathway. Future studies incorporating colony-level deep phenotyping, longitudinal population-genomic approaches, and functional perturbation of mannan-biosynthetic genes will be critical to delineate causal drivers of CWR and tolerance. Moreover, identifying the host receptors and signaling pathways that mediate altered recognition, polarization, and metabolic reprogramming will help unravel how immune pressure shapes fungal adaptation and may reveal immunomodulatory strategies to enhance antifungal therapy.

Together, our results demonstrate that *C. parapsilosis* can adapt simultaneously to host immune pressure and echinocandin therapy through complex CWR and transcriptional remodeling. These adaptations maintain virulence, enhance biofilm formation, and promote echinocandin tolerance—but create exploitable susceptibilities to AMB. Understanding the molecular basis of mannan-centric CWR and its effects on immune recognition may inform host-directed strategies and help optimize antifungal regimens for persistent *Candida* infections.

## Data Availability

WGS raw data have been deposited in the NCBI Sequence Read Archive (SRA) database under accession number PRJNA1269271. RNA-seq raw and normalized data sets have been deposited in the NCBI Gene Expression Omnibus (GEO) database under accession number GSE298776. All other data supporting the findings of this study are included in the article and its supplemental material.

## AFTER EPUB

*[This article was published on 13 February 2026 with incorrect spelling of the author, Diego Fuentes, and errors in a figure and the text. The errors were corrected in the current version, posted on 17 March 2026.]*

## SUPPLEMENTAL MATERIAL

Supplemental material spectrum.03043-25-s0001.pdf Supplemental text and Figures S1 to S5.

Table S1 spectrum.03043-25-s0002.xlsx Minimum inhibitory concentrations (MICs) (µg/ml) and the amino acid sequence data of ERG11 and FKS1.

Table S2 spectrum.03043-25-s0003.xlsx Variants that are not shared across all strains, with their corresponding functional annotation.

Table S3 spectrum.03043-25-s0004.xlsx Variants changing in each of the Cp10-Cp12 samples compared to Cp9.

Table S4 spectrum.03043-25-s0005.xlsx Variants lost in all later isolates (Cp10-12) compared to Cp9.

Table S5 spectrum.03043-25-s0006.xlsx Simplified version of variants lost in all isolates.

Table S6 spectrum.03043-25-s0007.xlsx Variants that are different between pairs of X,Y isolates, affecting genes that are DE between biofilm-vs-planktonic in a way that is different between the same X,Y isolates.

Table S7 spectrum.03043-25-s0008.xlsx Log2 fold change values of Cp10-12 compared to Cp9 in the planktonic condition.

Table S8 spectrum.03043-25-s0009.xlsx Log2 fold change values of Cp10-12 compared to Cp9 in the biofilm condition.

Table S9 spectrum.03043-25-s0010.xlsx Log2 fold change values of strains Cp9-12 in the biofilm condition compared to the planktonic condition.

ASM does not own the copyrights to Supplemental Material that may be linked to, or accessed through, an article. The authors have granted ASM a non-exclusive, world-wide license to publish the Supplemental Material files. Please contact the corresponding author directly for reuse.
