# Supplementary material for: Profound cell wall remodeling in Candida parapsilosis during systemic infection confers simultaneous tolerance to echinocandins and host immunity
Source: Microbiol Spectr. 2026 Feb 13;14(4):e03043-25. doi: 10.1128/spectrum.03043-25 (PMC13055393; doi:10.1128/spectrum.03043-25)
Supplement: Supplemental material — Supplemental text and Figures S1 to S5. [file spectrum.03043-25-s0001.pdf]

Supplemental Material

Variant calling

We used perSVade (v0.8) (50) to call and filter variants from raw sequencing data for each strain. The pipeline was run on paired-end reads using the following command:

perSVade.py --ref <reference genome> --threads 24 -o <output directory> -f1 <reads FWD> -f2 <reads RV> --mitochondrial_chromosome mito_C_parapsilosis_CDC317 -gff <gff> --run_smallVarsCNV --caller all --coverage 20 --mitochondrial_code 4 --gDNA_code 12 --ploidy 2 --remove_smallVarsCNV_nonEssentialFiles --min_chromosome_len 100000 --verbose --nvars 50 --nsimulations 2 --simulation_ploidies auto --range_filtering_benchmark theoretically_meaningful --min_CNVsize_coverageBased 600 --window_size_CNVcalling 300 --cnv_calling_algs HMMcopy,AneuFinder --skip_repeat_analysis --QC_and_trimming_reads

This pipeline first performed read preprocessing. Raw reads were trimmed with Trimmomatic (v0.38) (51) using default parameters, followed by FastQC (v0.11.9) (https://www.bioinformatics.babraham.ac.uk/projects/fastqc/) for quality assessment. Trimmed reads were then aligned to the *C. parapsilosis* CDC317 reference genome (version s01-m03-r49, CGD (52) using bwa mem (v0.7.17) (https://bio-bwa.sourceforge.net/bwa.shtml). Alignments were processed with samtools (v1.9) (53) and duplicate reads were marked with GATK MarkDuplicatesSpark (v4.1.2.0).

Small variants (SNPs and INDELs) were called assuming diploidy (--ploidy 2) with three independent callers: freebayes (v1.3.1), GATK HaplotypeCaller (v4.1.2.0) (54), and bcftools call (v1.9). Variants with a minimum coverage of 20 (--coverage 20) were retained as high-confidence (PASS) calls, applying best-practice filters specific to HaplotypeCaller and freebayes (50). The pipeline then normalized and merged calls into a consensus set (variant_calling_ploidy2.tab). This file includes three key metrics:

- NPASS – the number of algorithms supporting a variant with PASS filters,
- mean_fractionReadsCov_PASS_algs – mean fraction of reads supporting the alternative allele across PASS algorithms,
- common_GT – consensus genotype across callers (0/1 for heterozygous, 1/1 for homozygous).

Structural variants (SVs) were identified using two complementary approaches:

1. Copy number variants (CNVs) – deletions and duplications were inferred from read-depth changes across 300-bp windows using AneuFinder (v1.18.0) (55) and HMMcopy (v1.32.0) (56). CNVs spanning ≥600 bp (≥2 windows) were retained. Although some CNVs were initially detected, manual inspection in IGV (57) and read-depth visualization indicated that most arose from noisy coverage; therefore, CNVs were not further analyzed. Instead, we relied on per-gene coverage values obtained with mosdepth (v0.2.6) (58).
2. Breakpoint-resolved SVs – inversions, translocations, insertions, deletions, and tandem duplications were called based on split-read and discordant read-pair evidence, combined with local assembly. We used gridss (v2.9.2) (59) and clove (v0.17) (60), with isolate-

specific filtering parameters optimized automatically (--nvars 50 --nsimulations 2 --simulation_ploidies auto --range_filtering_benchmark theoretically_meaningful). This method allows precise identification of SV breakpoints. All SVs reported in this study refer to these breakpoint-defined variants.

Finally, both small variants and SVs were annotated using the Ensembl Variant Effect Predictor (VEP, v100.2) (61), which provided functional consequences including protein-altering variants (recorded in the column is_protein_altering). Annotation was performed with the GFF file corresponding to the CDC317 reference genome (s01-m03-r49) and appropriate translation codes for *C. parapsilosis* (--mitochondrial_code 4, --gDNA_code 12).

In summary, perSVade was used to generate filtered and annotated sets of SNPs, INDELs, and SVs, along with per-gene coverage estimates.

Variant filtering and integration

To combine small variants, gene coverage measurements, and functional annotations, we used the function get_integrated_small_vars_df_severalSamples from the module sv_functions.py in the perSVade package (https://github.com/Gabaldonlab/perSVade). This function generates three integrated tables, pooling data from all perSVade runs:

1. smallVars.tab – contains all small variants called across isolates, including information on other isolates where each variant was detected (other_samples_with_variant_called).
2. smallVars_annot.tab – provides functional annotations for small variants, ensuring that affected genes correspond to those defined in the supplied GFF file.
3. merged_coverage.tab – includes median gene coverage values for each isolate.

To obtain a high-confidence set of small variants for each isolate, we applied the following filters:

- NPASS ≥ 2
- mean_fractionReadsCov_PASS_algs ≥ 0.25
- Homozygous variants (common_GT = 1/1) required mean_fractionReadsCov_PASS_algs ≥ 0.9
- Heterozygous variants (common_GT = 0/1) were retained only if: i) common_GT = 0/1, ii) mean_fractionReadsCov_PASS_algs ≥ 0.25, and iii) mean_fractionReadsCov_PASS_algs ≤ 0.8.

Filtering was implemented using Python (v3.10.6) (62) and pandas (v1.5.1) (63).

For structural variant (SV) integration, we used the function get_integrated_SV_CNV_df_severalSamples from *sv_functions.py* in perSVade. This function generates two additional integrated tables:

1. SV_CNV.tab – contains all filtered SVs across isolates, with information on overlapping SVs in other isolates. Overlaps were defined as ≥75% reciprocal overlap with breakpoints

within ≤50 bp of each other (overlapping_samples_byBreakPoints_allCalled). The file also includes variantID_across_samples, which groups equivalent SVs with slightly different breakpoints.

2. SV_CNV_annot.tab – contains functional annotations of SVs, ensuring affected genes align with the GFF annotation. This table also includes the field is_transcript_disrupting, indicating whether an SV interrupts transcript structure.

To refine the SV calls, we applied additional high-confidence filters, following the strategy in (64). Specifically, we calculated variant allele frequency (VAF) for each breakend (https://github.com/PapenfussLab/gridss/issues/234) and excluded variants with low VAF that may represent spurious rearrangements. SVs were retained only if:

- at least one breakend had VAF ≥ 0.3, and
- all breakends had VAF ≥ 0.1.

All SV filtering and integration steps were performed using Python and pandas.

Comparison of variants across isolates

A key consideration in our variant analysis was how to compare variant sets between isolates. Although the isolates were clonal, they also showed divergence from the reference genome (Figures 1B-D), necessitating a tailored approach for cross-isolate comparisons. Variant calling can introduce errors (false positives), so for each isolate we applied stringent quality filters (see "Variant filtering and integration") to generate a "high confidence" set of variants. Only these variants were considered true for downstream analyses. However, because filtering itself can exclude genuine variants (false negatives), this creates challenges for comparisons across isolates. For example, when defining SNPs unique to Cp10 relative to Cp9, a straightforward strategy would be to select SNPs present in the high-confidence Cp10 set but absent from the high-confidence Cp9 set. Yet, this approach risks misclassifying some Cp10-specific SNPs, since variants shared between the two isolates could fail to meet the filtering threshold in Cp9. Given that these isolates differ from the reference genome by thousands of variants – most of them shared – even a small filtering error rate could produce an inflated number of false positive variants.

To minimize false positives, we applied a custom approach to identify variants gained or lost in a given target isolate relative to a set of background isolates (Tables S3–6). These variants were defined as "Private variants" using overlap information from the columns *other_samples_with_variant_called* (for small variants) and *overlapping_samples_byBreakPoints_allCalled* (for SVs), which consider all called variants/breakpoints rather than only high confidence ones. For SVs, the *variantID_across_samples* column was used as the sample-specific variant identifier.

We classified private variants into four categories:

1. Gained variants – called with high confidence in the target isolate and not overlapping any background isolates.

2. Lost variants – present in all background isolates with high confidence, but absent in the target isolate.

3. Loss-of-heterozygosity (LoH) variants – called with high confidence in the target and background isolates, but with the target sample showing *common_GT=1/1* while all background isolates showed *common_GT=0/1*.

4. Gain-of-heterozygosity (GoH) variants – similar to LoH, but with the target sample showing *common_GT=0/1* while all background isolates showed *common_GT=1/1*.

All filtering was performed using Python and pandas. This approach was used to identify variants present in isolates Cp10–Cp12 (targets) compared with the likely parental isolate Cp9 (background) (see Results; Tables S3 and S6; Figure 1E). For variants shared among Cp10–Cp12 (Tables S4 and S5), we manually inspected alignments in IGV, which led us to exclude *Contig005806_C_parapsilosis_CDC317_798154_T/TCCCCC*.

For broader presence/absence patterns across isolates (Table S2, Figure 1D), we used a slightly different approach. First, we retained only variants that were high confidence in some—but not all—isolate(s), using *variantID_across_samples* as the identifier for SVs. Next, the *other_samples_with_variant_called* and *overlapping_samples_byBreakPoints_allCalled* columns were used to determine, for each high-confidence variant, which isolates carried the variant, even if it failed filters in some cases. Variants inferred in this way but not passing filters were considered low confidence for the corresponding isolate.

Finally, to focus on functional relevance, we retained small variants predicted to alter protein sequences (*is_protein_altering*) and SVs predicted to disrupt transcripts (*is_transcript_disrupting*).

To generate functional annotations for each gene (reported in the supplementary tables), we integrated multiple sources of information.

1. CGD annotations – We retrieved chromosomal feature annotations for *C. parapsilosis* CDC317 from CGD (version s01-m03-r45; http://www.candidagenome.org/download/chromosomal_feature_files/C_parapsilosis_CDC317/archive/), consistent with the GFF file used. These annotations provided gene names, *Saccharomyces cerevisiae* orthologs, genomic coordinates, and functional descriptions.

2. Selection scores – We incorporated selection scores reported for other *Candida* species (Table S2 in (64); sheets “Gene_features” and “Selection_scores_all_genes”). Specifically, we mapped each *C. parapsilosis* gene to orthologs via the *orthofinder_orthocluster* column in the “Gene_features” sheet, and then linked each gene to *selection_score_S* and *significant_selection* values from the “Selection_scores_all_genes” sheet.

3. Gene Ontology (GO) terms – We added GO annotations from CGD (downloaded on 01/31/2022; http://www.candidagenome.org/download/go/gene_association.cgd.gz), retaining only annotations for *C. parapsilosis* CDC317 (taxon:578454). To remove obsolete terms, we updated GO annotations using the goatools.obo_parser.GODag object (v1.2.3) (65) with the GO basic .obo file (downloaded 31/01/2022; http://purl.obolibrary.org/obo/go/go-basic.obo). We then extracted GO terms by namespace: Biological Process (BP), Molecular Function (MF), and Cellular Component (CC), using Python and pandas.

4. Ortholog functional descriptions – We added descriptions from orthologs in other yeasts (downloaded 31/01/2022), including *C. albicans* (http://www.candidagenome.org/download/chromosomal_feature_files/C_albicans_SC5314/C_albicans_SC5314_version_A22-s07-m01-r177_chromosomal_feature.tab), *C. glabrata* (http://www.candidagenome.org/download/chromosomal_feature_files/C_glabrata_CBS138/C_glabrata_CBS138_version_s05-m01-r03_chromosomal_feature.tab), *C. auris* (http://www.candidagenome.org/download/chromosomal_feature_files/C_auris_B8441/C_auris_B8441_version_s01-m01-r25_chromosomal_feature.tab), and *S. cerevisiae* (http://sgd-archive.yeastgenome.org/curation/chromosomal_feature/SGD_features.tab). Ortholog mapping was performed using CGD orthology tables: *C_parapsilosis_CDC317_C_albicans_SC5314_by_CGOB* (for *C. albicans*), *C_parapsilosis_CDC317_C_glabrata_CBS138_by_CGOB* (for *C. glabrata*), *C_parapsilosis_CDC317_C_auris_B8441_by_CGOB* (for *C. auris*), and *C_parapsilosis_CDC317_S_cerevisiae_by_CGOB* (for *S. cerevisiae*).

Integration of RNA-seq and WGS highlights complex genetic and transcriptomic contributions to CWR and enhanced biofilm formation.

To explore why later isolates exhibit distinct biofilm properties, including biofilm-associated antifungal tolerance, we integrated whole-genome sequencing (WGS) and RNA-seq data. We hypothesized that strain-specific sequence variants may influence genes whose expression differs between planktonic and biofilm growth, thereby contributing to unique biofilm phenotypes.

For each isolate and timepoint (90 min, 8 h, 24 h), we first identified genes differentially expressed between planktonic and biofilm conditions (|log2 fold-change|>=1.5, pADJ < 0.05). We then performed pairwise comparisons between later isolates and Cp9 (Cp10 vs Cp9, Cp11 vs Cp9, Cp12 vs Cp9) to identify "distinct biofilm genes." These were defined as genes that: i) were differentially expressed in either isolate between planktonic and biofilm conditions, ii) carried a sequence variant present in only one of the isolates, and iii) showed substantially different differential expression between isolates (|log2 fold-change fgID - log2 fold-change bgID| >= 1.5 or absent expression in one isolate).

Using this approach, we identified three candidate genes (Supplementary Tables 6 and 9) with both sequence variation and distinct expression profiles:

1. *DUR3* – A variant in the 3' region, gained in Cp10, corresponded to higher upregulation in Cp10 (log2 FC = 2.6) compared to Cp9 (log2 FC = 0.43). *DUR3* encodes a transmembrane transporter.

2. CPAR2_600460 – A 5' variant gained in Cp11 was associated with unaltered expression in Cp11 (log2 FC = -0.06) versus upregulation in Cp9 (log2 FC = 1.65); this gene also encodes a transmembrane transporter.

3. *ALS7* – Three variants lost in Cp11 affected the coding sequence of this adhesin, a gene implicated in biofilm formation in *C. albicans*. *ALS7* was downregulated in Cp11 (log2 FC = -0.24) but upregulated in Cp9 (log2 FC = 1.72).

Although these candidate genes show combined genetic and transcriptional differences, the specific point mutations in these transporters or *ALS7* have not previously been linked to biofilm formation or cell wall remodeling. Nevertheless, this integrative analysis underscores that the enhanced CWR and biofilm formation observed in Cp11 and Cp12 likely arise from a complex interplay of genetic and transcriptomic changes, rather than from single mutations in canonical biofilm genes.

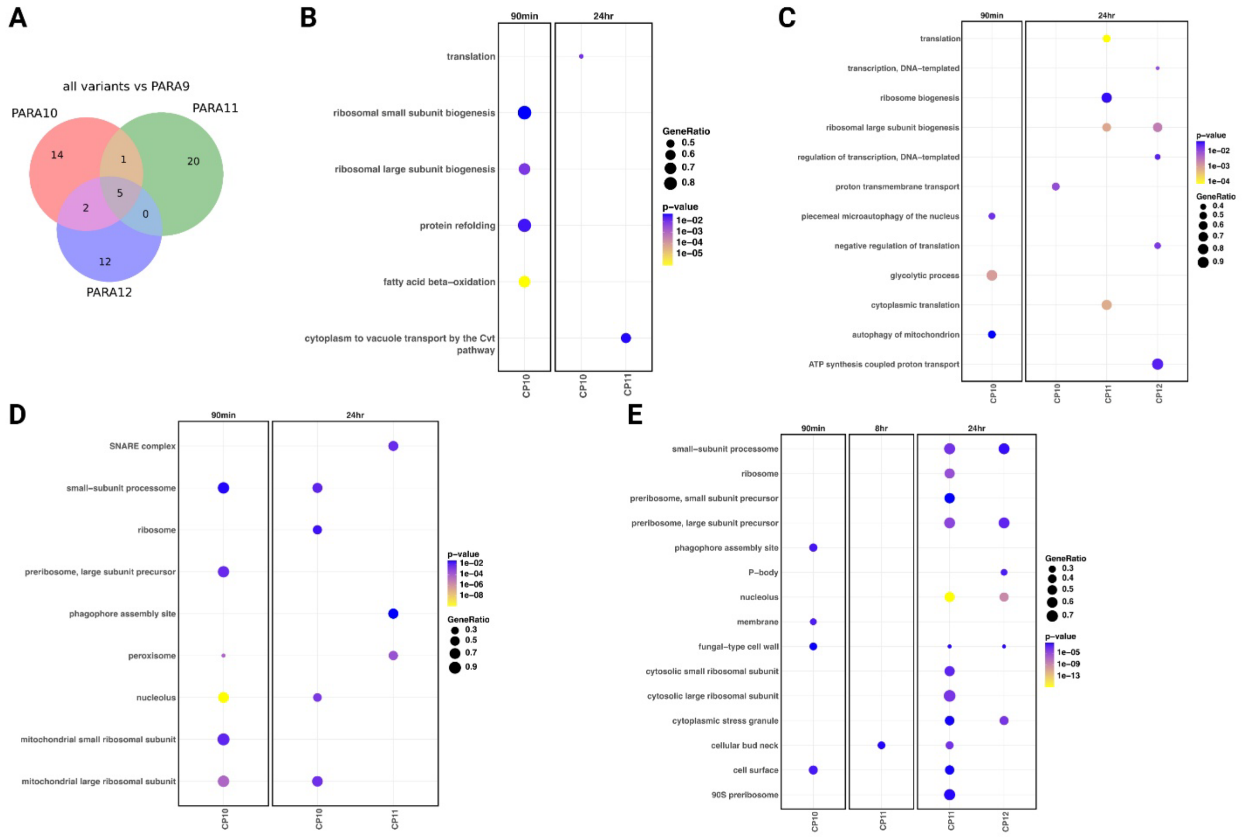

Figure S1. P**lots of *C. parapsilosis* isolates under planktonic conditions**. Venn diagram depicting the number of private variants in each sample as compared to PARA9 (Cp9) (A). Biological processes upregulated (B) and downregulated (C) by transcriptional profiling in Cp10-Cp12 relative to Cp9 at each timepoint in the planktonic condition. Log_2 fold change of gene expression in Cp10-Cp12 were obtained for each strain relative to Cp9 in each timepoint in the planktonic condition. Cellular components upregulated (D) and downregulated (E) by transcriptional profiling in Cp10-Cp12 relative to Cp9 at each timepoint in the planktonic condition. Log_2 fold change of gene expression in Cp10-Cp12 were obtained for each strain relative to Cp9 in each timepoint in the planktonic condition.

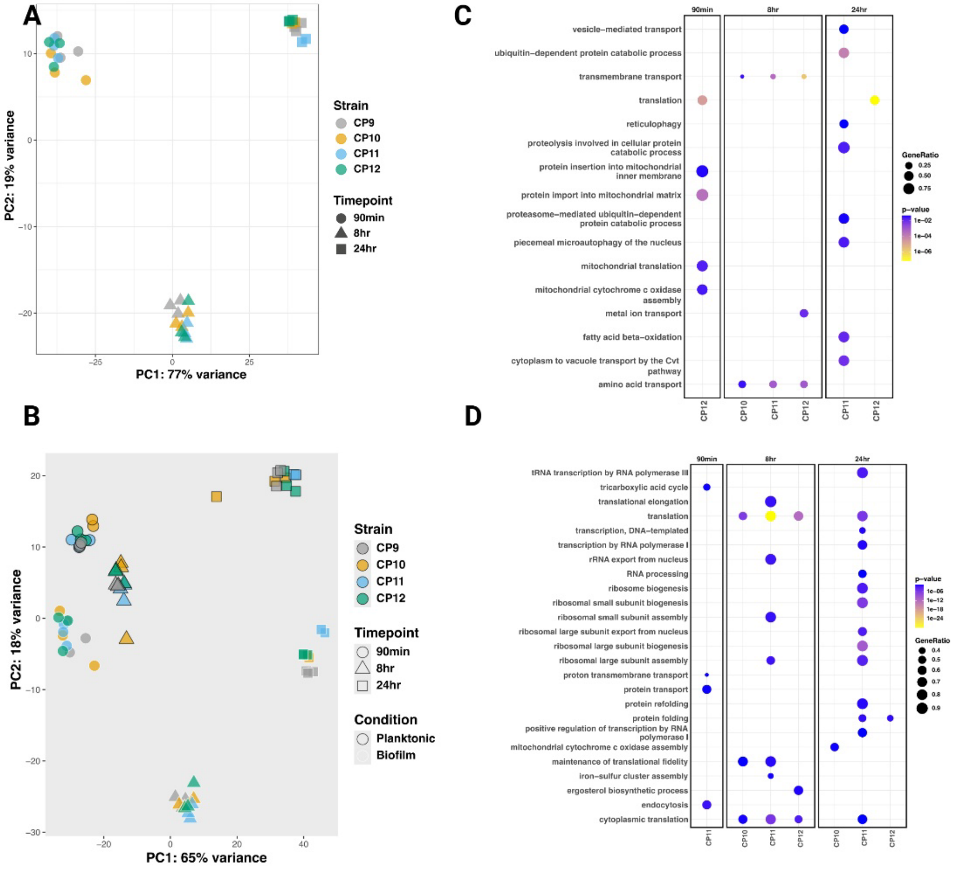

Figure S2. **Plots of *C. parapsilosis* isolates under biofilm conditions**. PCA plot depicting clustering of samples by RNA-Seq profiles across strains, timepoints and condition (A). PCA plot using all serial isolate samples in the three timepoints and two growth conditions (B). The x-axis shows the dimension with most variance computed using the top 500 genes that have the most variance across samples and the y-axis shows PC2, with the second most variation in the data. Biological processes upregulated (C) and downregulated (D) by transcriptional profiling in Cp10-12 relative to Cp9 at each timepoint in the biofilm condition. Log_2 fold change of gene expression in Cp10-Cp12 were obtained for each strain relative to Cp9 in each timepoint in the biofilm condition to identify strain-specific change in expression.

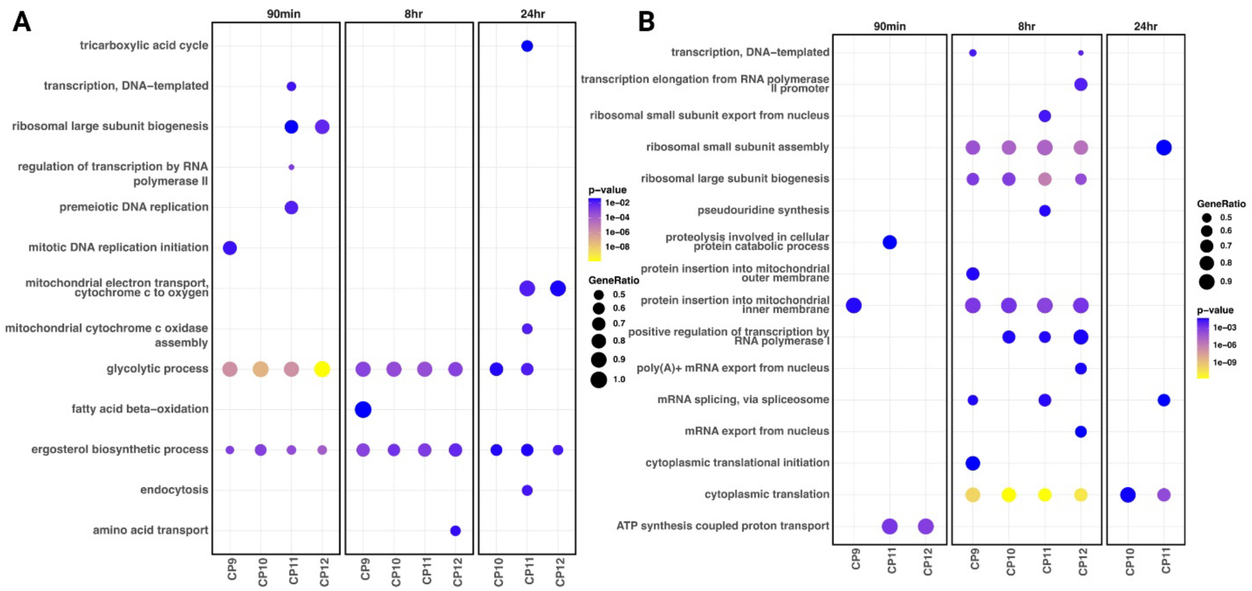

Figure S3. Biological processes differentially regulated in Cp9 or Cp10-Cp12 at each timepoint. Upregulated (A) and downregulated (B) biological processes by transcriptional profiling that are enriched either in the earlier isolate (Cp9) or at least in one of the later isolates (Cp10-Cp12) in at least one of the timepoints. The enriched functional categories were obtained using log2 fold change values in biofilm relative to planktonic conditions in corresponding strains and timepoints.

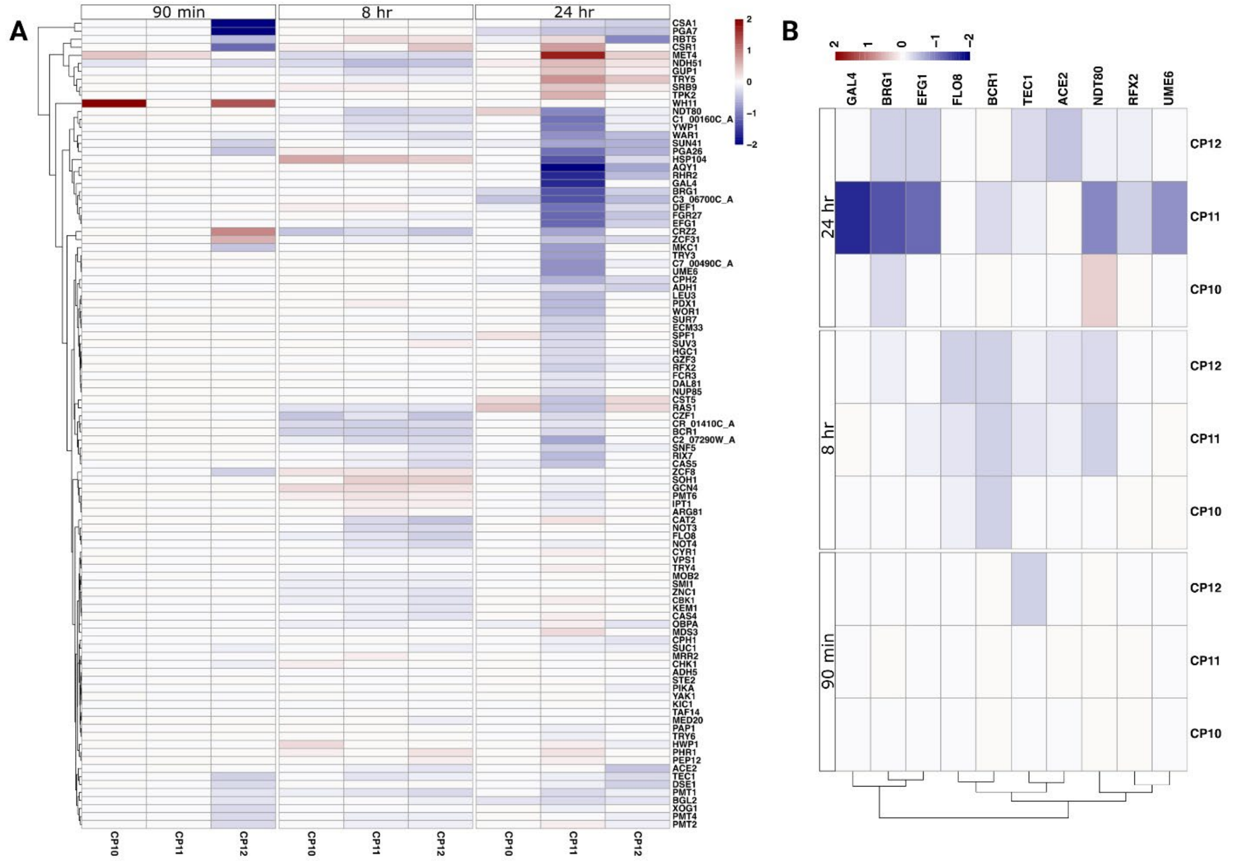

Figure S4. Expression over time of biofilm related genes in the biofilm condition across serial isolates Cp10-12 relative to Cp9. Biofilm-related genes in *C. albicans* were obtained based on the GO annotation "single-species biofilm formation on inanimate substrate" (A). A selection of known regulators of *C. albicans* biofilm formation and morphogenesis are depicted separately (B). If a corresponding ortholog is present in *C. parapsilosis*, it is included as a putative biofilm-related gene. The $\log_2$ fold change represents the change in expression in the later strains compared to Cp9 in the biofilm condition.

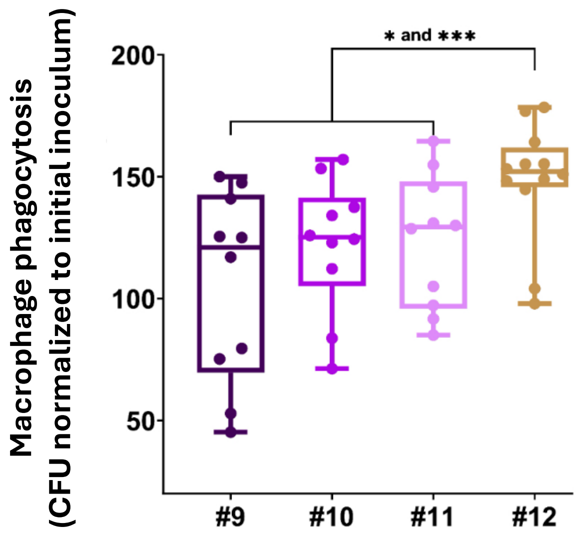

Figure S5. Phagocytosis by primary human macrophages using CFU counting. X-axis represents strain Cp9-Cp12, and Y-axis indicates CFUs phagocytosed. * $P \leq 0.05$ and *** $P \leq 0.001$. The Wilcoxon Signed Ranks Test was used for analysis.
